# Biomimetic black phosphorus quantum dots-based photothermal therapy combined with anti-PD-L1 treatment inhibits recurrence and metastasis in triple-negative breast cancer

**DOI:** 10.1186/s12951-021-00932-2

**Published:** 2021-06-13

**Authors:** Peiqi Zhao, Yuanlin Xu, Wei Ji, Shiyong Zhou, Lanfang Li, Lihua Qiu, Zhengzi Qian, Xianhuo Wang, Huilai Zhang

**Affiliations:** 1 Department of Lymphoma, Tianjin’s Clinical Research Center for Cancer, Key Laboratory of Cancer Prevention and Therapy, National Clinical Research Center for Cancer, Tianjin Medical University Cancer Institute and Hospital, Tianjin Medical University, 24 Huanhu West Road, Hexi District, Tianjin, 300060 People’s Republic of China; 2 Department of Lymphatic Comprehensive Internal Medicine, Affiliated Cancer Hospital of Zhengzhou University, Zhengzhou, 450001 Henan China; 3 Public Laboratory, Tianjin’s Clinical Research Center for Cancer, Key Laboratory of Cancer Prevention and Therapy, National Clinical Research Center for Cancer, Tianjin Medical University Cancer Institute and Hospital, Tianjin Medical University, Tianjin, 300060 China

**Keywords:** Anti-PD-L1, Biomimetic black phosphorus quantum dots, Photothermal immunotherapy, Triple-negative breast cancer, Tumor recurrence and metastasis

## Abstract

**Background:**

Triple-negative breast cancer (TNBC) is a highly aggressive malignant disease with a high rate of recurrence and metastasis, few effective treatment options and poor prognosis. Here, we designed and constructed a combined photothermal immunotherapy strategy based on cancer cell membrane-coated biomimetic black phosphorus quantum dots (BBPQDs) for tumor-targeted photothermal therapy and anti-PD-L1 mediated immunotherapy.

**Results:**

BBPQDs have good photothermal conversion efficiency and can efficiently target tumor cells through homologous targeting and tumor homing. Under near infrared irradiation, we found that BBPQDs kill tumors directly through photothermal effects and induce dendritic cells maturation. In vivo studies have confirmed that the combined photothermal immunotherapy strategy displays a stronger antitumor activity than anti-PD-L1 monotherapy. In addition, BBPQDs-mediated photothermal therapy in combination with anti-PD-L1 treatment inhibit tumor recurrence and metastasis by reprograming the immunosuppressive tumor microenvironment into an immune-active microenvironment, and promoting the local and systemic antitumor immune response. We further found that the combined photothermal immunotherapy strategy can produce an immune memory effect against tumor rechallenge.

**Conclusions:**

This study provides a novel therapeutic strategy for inhibiting the recurrence and metastasis of TNBC, with broad application prospects.
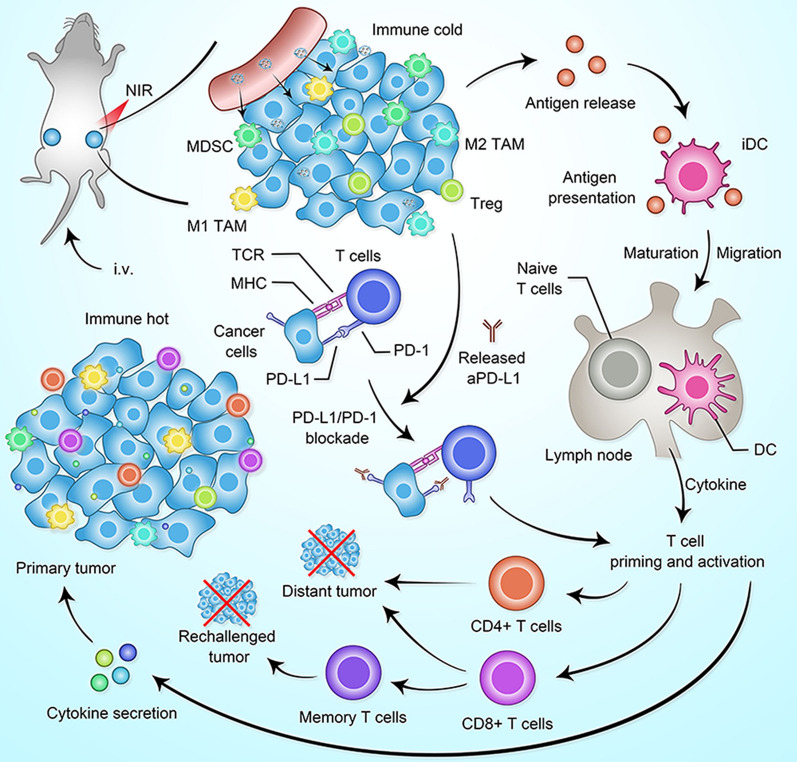

**Supplementary Information:**

The online version contains supplementary material available at 10.1186/s12951-021-00932-2.

## Introduction

Triple-negative breast cancers (TNBC) are those that lack expression of estrogen receptor, progesterone receptor, and human epidermal growth factor receptor 2 and account for ~ 15–20% of all breast cancers [1–3]. The most commonly used clinical treatments are surgery and chemotherapy. TNBC develops rapidly and is prone to recurrence and metastasis. The long-term efficacy of chemotherapy is poor [3–5]. The 5-year survival rate for patients with metastatic TNBC is less than 30% [6, 7]. Therefore, there is an urgent need for effective treatment strategies.

In recent years, immune checkpoint blockade (ICB) therapies have changed the paradigm of tumor treatment, but only 10–30% of patients with TNBC can achieve long-term durable remission; most patients do not respond significantly to ICB or remain resistant to it [8–10]. Reasons for ineffective treatment with ICB include a lack of specific antigens on the surface of tumor cells that can be recognized by immune cells, a lack of tumor infiltrating lymphocytes, an immunosuppressive tumor microenvironment (TME), or other suppressive immune checkpoints and suppressive cytokines [11, 12]. Therefore, designing a combination therapeutic strategy that promotes immune response, overcomes the immunosuppressive TME, and induces persistent immunity is expected to further improve the efficacy of ICB against TNBC and prevent tumor recurrence and metastasis [13–17].

Photothermal therapy (PTT) has become a novel and rapidly developing cancer treatment method, that offers low toxicity and high spatial selectivity compared to conventional cancer ablation methods [18–20]. PTT is a treatment method that kills cancer cells by injecting materials with a high photothermal conversion efficiency inside the organism, relying on targeted identification technology to gather near the tumor tissue, and converting light energy into heat energy by absorbing near-infrared (NIR) light [13, 21]. In addition to directly ablating tumor cells, PTT can also trigger antitumor immune response and inhibit tumor recurrence and metastasis [22, 23]. Because of the limited penetration depth of the NIR laser in the body, it is especially suitable for relatively superficial tumors such as breast cancer and head and neck tumors. Common photothermal converters include gold nanorods [24], Ag_2_S nanoparticles [25], graphene oxide [26], gold nanoshells [27], and Prussian blue nanoparticles [28].

Black phosphorus is a novel two-dimensional layered inorganic material with excellent properties such as high light absorption and photothermal conversion efficiency and good biocompatibility. It is widely used in PTT for tumors [29, 30]. Black phosphorus quantum dots (BPQDs) are prepared from black phosphorus and have efficient NIR photothermal effects for killing cancer cells and faster clearance [31]. However, BPQDs still have certain limitations, such as instability and poor targeting ability [32]. In recent years, cell membrane camouflage technology has been widely used to construct nano-drug formulations for tumor diagnosis and treatment [33]. Extensive research confirmed that the adhesion molecules expressed on cancer cell membranes can navigate and anchor cancer cells to each other through the formation of receptor-ligand binding [34, 35]. Inspired by this, coating BPQDs with a homologous cancer cell membrane can improve the stability, avoid the recognition and clearance of heterologous substances by immune cells, and make the BPQDs accumulate and colonize efficiently at the tumor site through the dual effects of homologous targeting and tumor homing [33, 36, 37].

**Scheme 1:**
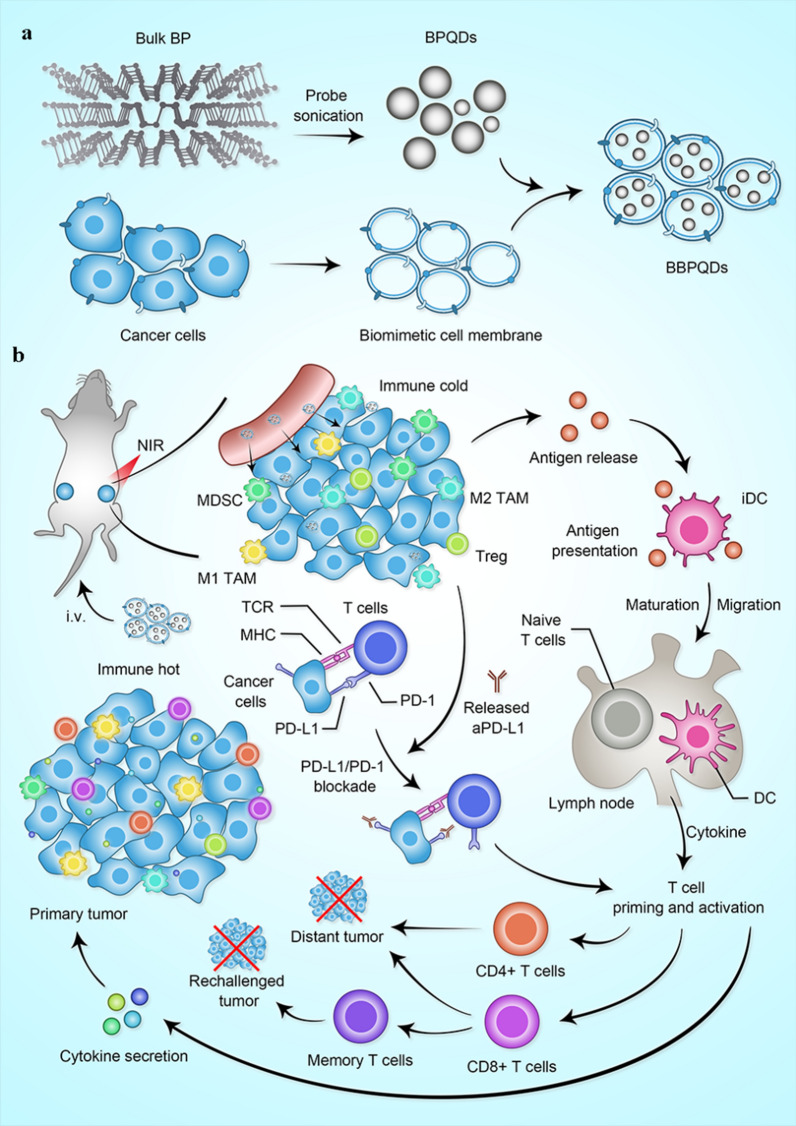
**a** Illustration of the synthesis process of BBPQDs. **b** Potential mechanism of BBPQDs-mediated PTT in combination with ICB therapy for antitumor treatment

In this work, cancer cell membrane-coated biomimetic BPQDs (BBPQDs) were designed and constructed for tumor-targeted PTT (Scheme 1). We found that BBPQDs have good photothermal conversion efficiency, which can not only kill tumors directly through photothermal effects, but also induce maturation of dendritic cells (DCs). In addition, anti-PD-L1(αPD-L1) treatment blocks PD-1/PD-L1 pathways and enhances the T-cell immune response. This enables T cells to recognize and kill tumor cells. BBPQDs-mediated PTT in combination with αPD-L1 immunotherapy inhibit tumor recurrence and metastasis by reprograming the immunosuppressive TME into an immune-active microenvironment, thus promoting the local and systemic antitumor immune response. We also found that this combination therapy strategy has an immune memory effect against tumor rechallenge. This study shows that the combined photothermal immunotherapy strategy improves the efficacy of TNBC, effectively inhibits the recurrence and metastasis, and has a wide range of applications in cancer treatment.

## Materials and methods

### Synthesis of BBPQDs

The BPQDs were prepared from bulk BP by sonication assisted liquid-phase exfoliation [38]. Cancer cell membranes were firstly prepared using a previous method [39]. To achieve cancer cell membrane encapsulation, BPQDs were mixed with cancer cell membranes at a weight ratio of 3:1, and the prepared cancer cell vesicles were mixed and resuspended in phosphate buffer solution (PBS). We then extruded the sample 11 times with an Avanti mini-extruder and centrifuged to remove the excess cancer cell vesicles. Finally, the prepared BBPQDs were placed in 1 × PBS at 4 °C for further use.

### In vitro photothermal imaging

The photothermal effects of PBS, BPQDs, and BBPQDs under 808 nm laser irradiation were investigated. Here, 2 mL of solutions containing BBPQDs with various concentrations were irradiated under 808 nm laser (1.0 W/cm^2^) for 5 min. The BBPQDs were also exposed to an 808 nm laser with different powers. The temperatures of these samples at different time points were measured every 30 s using an infrared thermal imaging camera.

To investigate the photothermal stability of BBPQDs solution (50 μg/mL), it was first irradiated under an 808 nm laser (1.0 W/cm^2^) for 5 min. The laser was turned off, and the temperature was allowed to cool naturally to room temperature without irradiation. The process of warming and cooling was repeated five times, and the temperature was recorded every 30 s.

### In vitro cytotoxicity

To exploit the photothermal cytotoxicity of BPQDs and BBPQDs, 4T1 cells (1 × 10^4^ cells per well) were seeded into 96-well plates and incubated at 37 °C for 24 h. Different concentrations of BPQDs and BBPQDs were then added into the wells, incubated with cells for 4 h, and then irradiated by the NIR laser (808 nm, 1.0 W/cm^2^) for 5 min. The cells were then incubated for another 24 h. Cell viability was determined using a MTT assay according to the manufacturer’s protocol.

### Apoptosis assessment by flow cytometry

Briefly, 1 × 10^5^ 4T1 cells were seeded in 6-well plates for 24 h. The cells were then incubated with BPQDs and BBPQDs for 4 h and then irradiated with a NIR laser (808 nm, 1.0 W/cm^2^) for 5 min. The cells were collected, stained with the Annexin V-FITC/PI Apoptosis Detection Kit for 15 min, and analyzed with flow cytometry.

### In vivo biodistribution of BBPQDs

Cy5.5 labeled BPQDs and BBPQDs were injected into tumor-bearing mice via the tail vein at a dose of 10 mg/kg. The in vivo imaging was performed with an IVIS imaging system at predetermined time intervals post injection. Tumors and major organs such as heart, liver, spleen, lung, and kidneys were harvested and photographed 48 h after intravenous injection.

### In vivo photothermal imaging

For in vivo photothermal imaging, BPQDs and BBPQDs were injected intravenously into tumor-bearing mice at a dose of 10 mg/kg when the tumors reached approximately 100 mm^3^ in size; 24 h later, the tumor sites were irradiated under a NIR laser (808 nm, 1.0 W/cm^2^) for 5 min, and the infrared thermal images were recorded with an infrared thermal camera. Mice injected with PBS were used as controls.

### In vivo DCs activation

A 4T1 subcutaneous tumor model was used to observe the effect of PTT on DCs maturation. When the tumors grew to 100 mm^3^, PBS, BPQDs + NIR irradiation and BBPQDs + NIR irradiation (808 nm, 1.0 W/cm^2^, 5 min) were administered. After irradiation, tumor-draining lymph nodes were collected and monodisperse cells were isolated and stained with anti-CD11c, anti-CD86 and anti-CD80, respectively. DCs maturation (CD11c^+^CD80^+^CD86^+^) was detected by flow cytometry.

### Therapeutic effect on tumors in situ

4T1-Luc cells (1 × 10^6^) were injected subcutaneously into the left flank of female mice to evaluate the efficacy of PTT in combination with αPD-L1 on primary tumors. When the tumor volume reached about 100 mm^3^, the tumor-bearing mice were randomly divided into five groups (n = 6 per group): PBS as the control group; αPD-L1, BPQDs with NIR irradiation; BBPQDs with NIR irradiation; and BBPQDs with NIR irradiation and αPD-L1. Then, 100 μL of therapeutic agents at a dose of 10 mg/kg were injected into the mice via the tail vein on day 0. At 24 h after injection, the tumor region was irradiated by the NIR laser (808 nm, 1.0 W/cm^2^) four times every three days for 5 min. The αPD-L1 was intraperitoneally injected into mice at a dose of 10 mg/kg four times every three days for αPD-L1 monotherapy or combination therapy. The tumor volume (0.5 × length × width^2^) and weight of mice were recorded every two days during the treatment period. Bioluminescence imaging was acquired through intraperitoneal injection of D-luciferin (150 mg/kg body weight, PerkinElmer). The survival time of mice was recorded from the day of tumor inoculation. The survival curve was plotted by Kaplan–Meier survival analysis. After treatment, tumor tissues and major organs of mice were taken, dehydrated, embedded, and sectioned for 3 μm. The sections were then stained with hematoxylin and eosin (H&E) and Ki-67 for histological analysis. The apoptosis of tumor cells was detected by TUNEL assays. The whole blood was harvested and then analyzed by a blood biochemistry analyzer (MNCHIP, China) and auto hematology analyzer (MC-6200VET) through the end of the experiments.

### Therapeutic effect on distant tumors

To evaluate the efficacy of the combined photothermal immunotherapy strategy on distant tumors, a bilateral subcutaneous tumor model was used in this study. First, 4T1 cells (1 × 10^6^) were subcutaneously inoculated on the left flank of the mice to simulate the primary tumor, and then on the right flank of the mice 3 days later to simulate the distant tumor. The mice were randomly divided into five groups (n = 6 per group) and treated with PBS, αPD-L1, BPQDs with NIR irradiation, BBPQDs with NIR irradiation, and BBPQDs with NIR irradiation and αPD-L1 when the primary tumor volume reached about 100 mm^3^. NIR irradiation (1.0 W/cm^2^, 5 min) was applied to the primary tumors four times every three days at 24 h post-injection. αPD-L1 (10 mg/kg) was injected intraperitoneally at 1, 4, 7, and 10 days after irradiation. The tumor volume of the distant tumors and weight of mice were recorded. The right flank tumors were collected to study the effects of the combination therapy on the TME. The percentages of T-cell infiltration, M1 phenotype macrophages (CD80^+^CD11b^+^F4/80^+^) and M2 phenotype macrophages (CD206^+^CD11b^+^F4/80^+^) in the distant tumors were analyzed by flow cytometry. T-cell infiltration and antitumoral cytokines like interleukin-6 (IL-6), tumor necrosis factor-α (TNF-α) and interferon-γ (IFN-γ) in distant tumors from each treatment were also monitored using CLSM and ELISA.

### Therapeutic effect on rechallenged tumors

To assess the effect of combination therapy on tumor recurrence and metastasis, we rechallenged mice previously treated with αPD-L1, BBPQDs with NIR irradiation, and BBPQDs with NIR irradiation and αPD-L1. The primary tumors were inoculated as above. When the tumors were 100 mm^3^, the mice were randomly divided into three groups (n = 6 per group) and treated as described above. The treatment was repeated four times at an interval of 7 days. At day 30, 4T1 tumors were inoculated by injecting 1 × 10^5^ cells into the tail veins in mice. The rechallenged tumors in lung were recorded and imaged at day 45. Tumor metastasis sites subsequently appeared as white nodules on the surface of the lungs and were also examined by H&E staining. To investigate the effect of the combination therapy on antitumor immunological memory, effector memory T cells (T_EM_, CD3^+^CD8^+^CD44^+^CD62L^−^) in spleen were analyzed by flow cytometry at day 33.

### Statistical analysis

All experiments were repeated at least three times unless otherwise specified. All experimental results are presented as mean ± standard deviation. The statistical differences between groups were calculated by Tukey’s test. *P < 0.05 was considered as statistically different and **P < 0.01 was considered to be significantly different.

## Results and discussion

### Preparation and characterization of BBPQDs

**Fig. 1:**
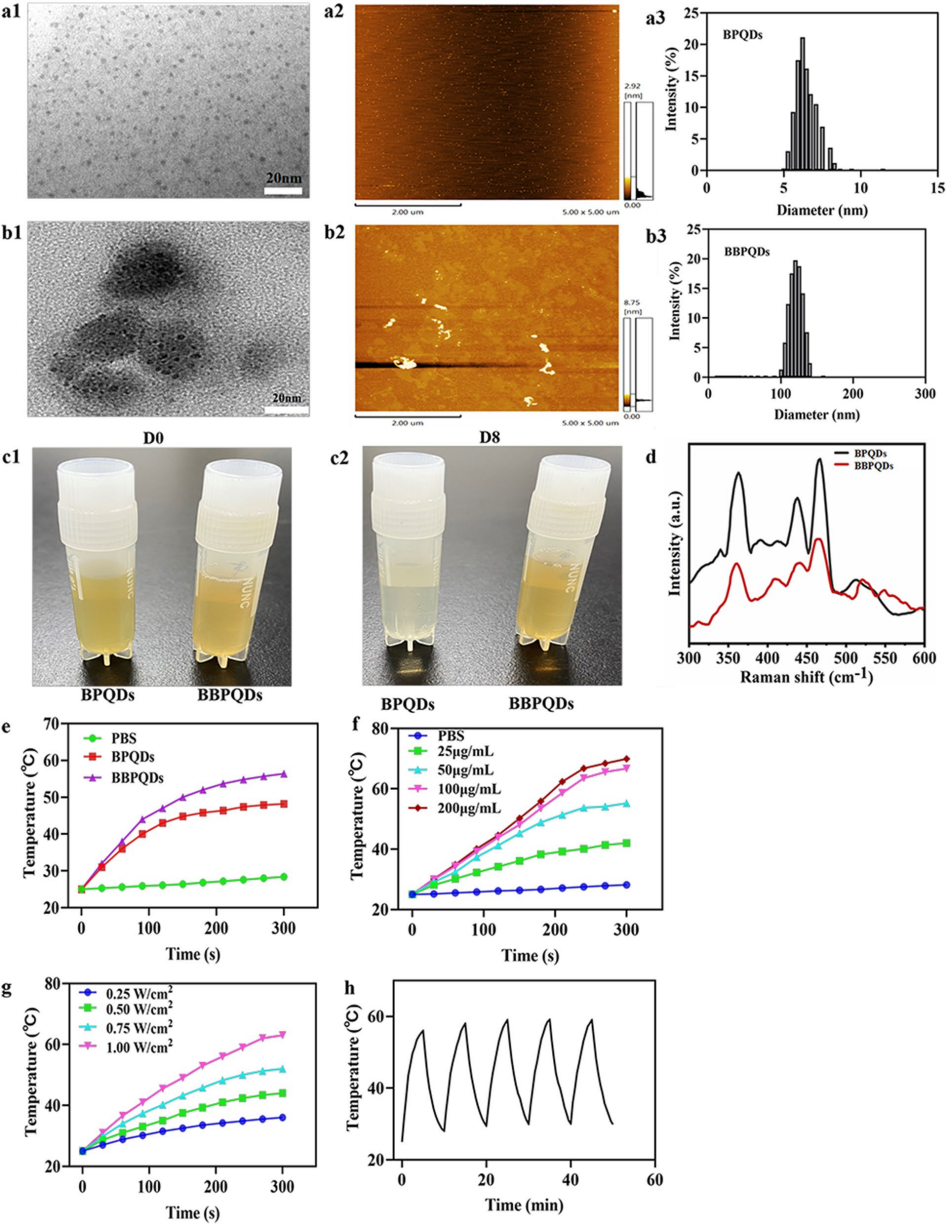
Characterization of BPQDs and BBPQDs. **a1** TEM analysis of BPQDs. **a2** AFM analysis of BPQDs. **a3** Size distributions of BPQDs determined by DLS. **b1** TEM analysis of BBPQDs. **b2** AFM analysis of BBPQDs. **b3** Size distributions of BBPQDs determined by DLS. **c** Photographs of the BPQDs and BBPQDs stored for different periods of time (0 and 8 days). **d** Raman scattering spectra acquired from the BPQDs and BBPQDs. **e** Heating curves of PBS, BPQDs, and BBPQDs upon 808 nm laser irradiation (1.0 W/cm^2^).** f** Heating curves of BBPQDs at different concentrations under laser irradiation (808 nm, 1.0 W/cm^2^). **g** Heating curves of BBPQDs at different power densities. **h** Heating curves after five cycles of 808 nm laser exposure (1.0 W/cm^2^)

The synthetic process of BBPQDs is shown in Scheme 1a. We applied sonication liquid exfoliation technique to prepare BPQDs, and then wrapped cancer cell membranes on their surface to prepare BBPQDs capable of escaping the host immune system and homologous targeting. At the same time, the encapsulation of cancer cell membrane improved the stability of BBPQDs, active targeting and enrichment ability of BBPQDs in tumors. TEM and AFM were employed to characterize the surface morphology of the BPQDs and BBPQDs (Fig. 1a, b). The average size of the BPQDs was about 3 nm (TEM), and the average height of the BPQDs was about 1–2 nm (AFM). For BBPQDs, the average size was 30 nm (TEM), and the average height was about 3–4 nm. The average diameters of the BPQDs and BBPQDs measured by TEM were smaller than the dynamic light scattering (DLS) measurements. This is because the diameter obtained from the DLS experiments reflects the hydrodynamic diameter of the nanoparticles, whereas the particle size observed by TEM reflects the diameter of the dried nanoparticles. Similar differences in particle size due to different measurement techniques were also previously reported [40, 41]. The particle size of BBPQDs is larger than that of BPQDs because multiple BPQDs are encapsulated within one cancer cell membrane.

The zeta potential of the BPQDs and BBPQDs was also investigated (Additional file 1: Fig. S1). BPQDs have zeta potential values of − 34.1 ± 4.0 mV whereas BBPQDs showed lower values of − 24.1 ± 3.1 mV, which are close to those of cancer cell membrane (− 19.9 ± 3.0 mV). Considering that proteins on the cell membrane play an important role in homologous targeting and immune escape of tumor cells, three major membrane proteins (CD47, gp100 and Pan-Cadherin) were investigated by Western blotting. Results showed that CD47, gp100 and Pan-Cadherin were significantly enriched on BBPQDs, indicating that the successful transfer of the membrane proteins to the shell of the nanoparticles (Additional file 1: Fig. S2).

To assess the effect of cancer cell membrane encapsulation on the stability of BPQDs, BPQDs and BBPQDs with the same concentration were dispersed in water and exposed to air for 8 days (Fig. 1c). Results showed that the color of BPQDs became lighter after 8 days, whereas the color of the BBPQDs solution remained unchanged. Furthermore, BPQDs and BBPQDs are also dispersed in the RPMI 1640 medium containing 10% FBS for 8 days to examine their stability in biological environment by DLS. The results also demonstrated that the BBPQDs exhibit a good stability (data not shown). Our results are consistent with previous findings that cancer cell membranes play a key role in stabilizing nanoparticles and protecting the internal content [42, 43].

The BPQDs and BBPQDs were also characterized using Raman spectroscopy (Fig. 1d). In BPQDs, three prominent Raman peaks can be observed; these were caused by an out-of-plane phonon mode A^1^g located at 361.5 cm^−1^ and two in-plane modes, B_2_g and A^2^g, located at 437.3 and 464.5 cm^−1^, corresponding to their theoretical calculated frequencies at 360, 440, and 470 cm^−1^, respectively [42]. Compared to BPQDs, the A^1^g, B_2_g, and A^2^g modes of BBPQDs were blue-shifted by about 1.4, 0.6, and 2.6 cm^−1^, respectively. When cancer cell membrane was coated on the surface of BPQDs, the oscillation of P atoms of BPQDs was maybe hindered to some extent, leading to the decrease of corresponding Raman scattering energy. As shown in Additional file 1: Fig. S3, the XRD pattern of BPQDs and BBPQDs exhibit three similar peaks at 17.0°, 34.1°, and 52.1°, respectively corresponding to (020), (040), and (060) planes, which demonstrate an orthorhombic crystal of BPQDs.

### In vitro cellular uptake

In order to evaluate the homologous targeting and immune escape effects of BBPQDs, we applied 4T1 and RAW 264.7 cells to observe the cellular uptake behavior of BPQDs and BBPQDs. For 4T1 cells (Additional file 1: Fig. S4), the internalization efficiency of FITC labeled BBPQDs was much higher than that of FITC labeled BPQDs, indicating the good targeting efficiency after the cancer cell membrane coating. However, RAW 264.7 cells exhibited brighter green fluorescence after being incubated with BPQDs, while for BBPQDs, fluorescence intensity was nearly negligible (Additional file 1: Fig. S5), reflecting the concept that cancer cell membrane coating effectively suppressed the macrophage engulfment and exhibited the favorable immune evasive efficacy.

### In vitro photothermal behavior of BBPQDs

BBPQDs can strongly absorb NIR light and convert it into thermal energy; thus, we investigated the photothermal conversion efficiency of BPQDs and BBPQDs under 808 nm laser irradiation (1.0 W/cm^2^, 5 min). Versus PBS, where the temperature change was small, significant temperature rises of 23.2 and 31.4 °C were observed for BPQDs and BBPQDs solutions, respectively, at a concentration of 50 μg/mL (Fig. 1e). The higher photothermal conversion efficiency of BBPQDs compared to BPQDs is due to the wrapping of the cancer cell membrane reducing the degradation of BPQDs and the wrapping of multiple BPQDs within a single cancer cell membrane. Moreover, the temperature rise of the BBPQDs was concentration, irradiation power, and time- dependent (Fig. 1f, g). In addition to their good photothermal conversion capability, BBPQDs also have high photostability and good reproducibility (Fig. 1h). These results suggest that BBPQDs can be used as an effective photothermal agent in PTT.

### In vitro cytotoxicity

**Fig. 2:**
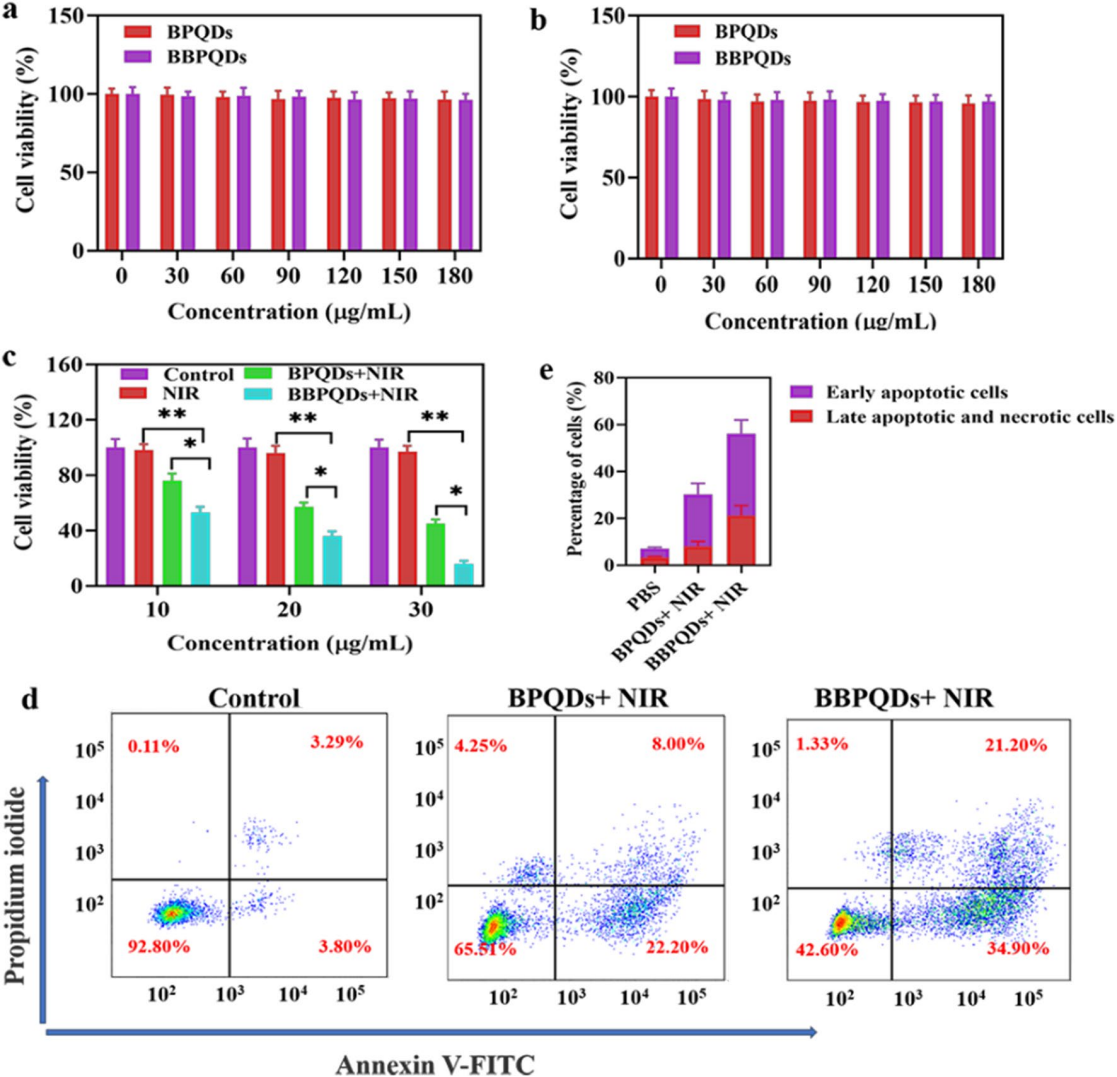
Therapeutic efficacy of BBPQDs in vitro. **a** Cell viability of HBL-100 cells treated with BPQDs and BBPQDs*.* **b** Cell viability of 4T1 cells treated with BPQDs and BBPQDs. **c** Cell viability of 4T1 cells treated with NIR, BPQDs + NIR, and BBPQDs + NIR (808 nm, 1.0 W/cm^2^, 5 min). **d** Flow cytometric analysis of 4T1 cells apoptosis induced by different formulations under irradiation (808 nm, 1.0 W/cm^2^, 5 min) or not using Annexin V-FITC PI staining. **e** Corresponding percentages of early apoptotic and late apoptotic tumor cells after different treatments using flow cytometry analysis

Excellent biocompatibility is a prerequisite for the clinical applications of nanocarriers. We next investigated the biosafety of BPQDs and BBPQDs. These results showed that the safety of BPQDs and BBPQDs was very good without NIR irradiation, and there was no significant cytotoxicity in HBL-100 and 4T1 cells even at a concentration of 180 μg/mL (Fig. 2a, b). In contrast, the cell killing efficiency increased with increasing concentrations of BPQDs and BBPQDs under NIR laser irradiation, and the cell survival rate was only 16.01% when the BBPQDs concentration reached 30 μg/mL. BBPQDs exhibited a stronger cytotoxicity effect for the 4T1 cells at the same concentration under NIR irradiation (Fig. 2c). Flow cytometry was used to detect the cell death mechanism after different treatments using the Annexin V-FITC/PI method. Figure 2d, e show no obvious early apoptosis or late apoptosis in the group treated by PBS. On the contrary, when the cells were treated with BPQDs under NIR irradiation, early and late apoptotic cells increased to 22.20% and 8.00%, respectively. The proportions of early apoptotic and late apoptotic and necrotic cells were more obvious in the group of cells treated with BBPQDs under NIR irradiation, which was calculated to be 34.90% and 21.20%. These results showed that wrapping of cancer cell membranes on the surface of BPQDs under NIR irradiation can enhance the PTT effect by promoting cell apoptosis.

### In vivo biodistribution

**Fig. 3:**
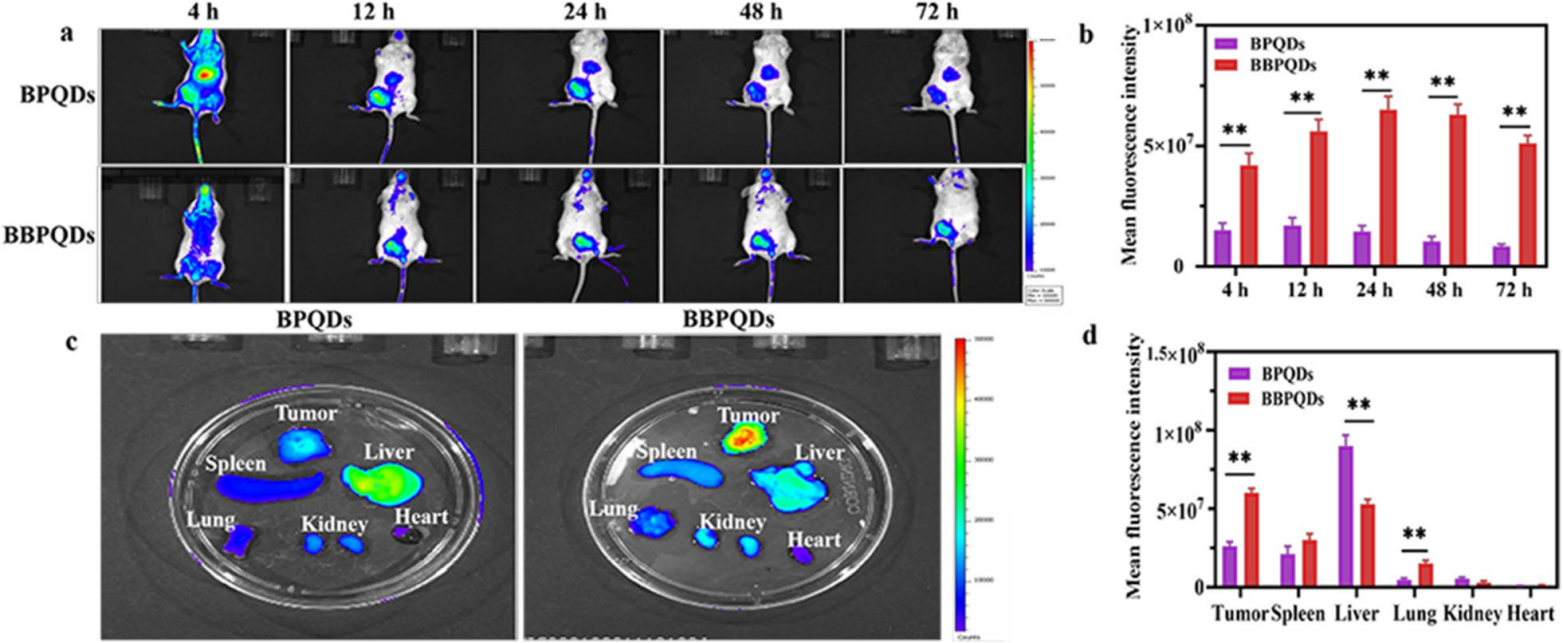
In vivo biodistribution of BBPQDs. **a** In vivo fluorescence images of representative 4T1 tumor-bearing mice after the injection of Cy5.5-labeled BPQDs and BBPQDs for different time intervals. **b** Quantitative fluorescence intensity statistics of tumor sites at different times. **c** Ex vivo imaging showing the distribution of Cy5.5-labeled BPQDs and BBPQDs in the tumor tissues and major organs at 48 h post injection. **d** Quantitative analysis of the Cy5.5 fluorescence intensity in the tumors and major organs

To investigate the in vivo tumor targeting and tissue distribution of BBPQDs, BALB/c mice bearing 4T1 tumors were injected intravenously with Cy5.5-labeled BPQDs and BBPQDs. The results showed that the fluorescence intensity of the BBPQDs group was significantly higher than that of the BPQDs group. In addition, the BBPQDs group had the strongest fluorescence intensity at 24 h post injection. Notably, the fluorescence intensity of the BBPQDs group was still very strong even 72 h after injection, thus showing good tumor targeting, high aggregation, and good retention of BBPQDs at the tumor tissue (Fig. 3a, b).

Mice were sacrificed 48 h after injection, and the tumors and major organs were dissected, followed by in vivo imaging. The results showed that the signals of the BBPQDs were stronger in tumors than in other organs, indicating that BBPQDs had better tumor targeting efficiency in vivo (Fig. 3c, d). BPQDs were selectively distributed in tumor tissues due to the enhanced permeability and retention effect (< 200 nm). Notably, the enrichment of liver in the BBPQDs group was lower than that of BPQDs, indicating that the BBPQDs wrapped by cancer cell membrane have good immune escape ability in vivo. The above results further showed the degree to which BBPQDs can perform homologous targeting.

### In vivo photothermal evaluation

**Fig. 4:**
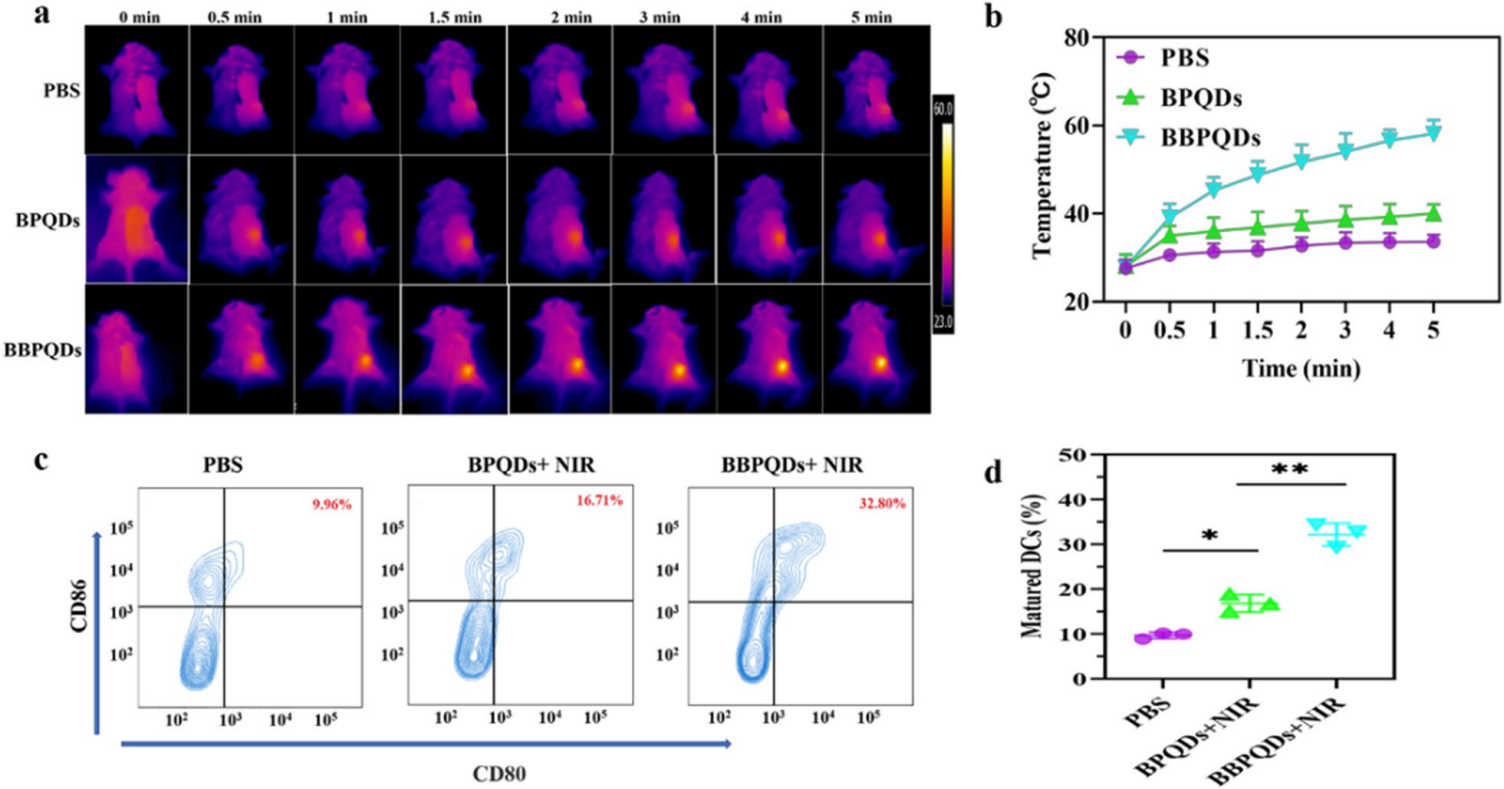
Prepared BBPQDs-mediated PTT induces the activation of DCs in vivo. **a** Infrared thermographic images of tumor bearing mice with various treatments after NIR irradiation (808 nm, 1.0 W/cm^2^, 5 min). **b** Temperature changes in irradiated areas of tumor bearing mice with various treatments were measured during NIR irradiation (808 nm, 1.0 W/cm^2^, 5 min). **c** Representative flow cytometry data to show DCs maturation in lymph nodes induced by different treatments in vivo. **d** Representative statistical data to show DCs maturation in lymph nodes induced by different treatments in vivo

To evaluate the photothermal effect of BPQDs and BBPQDs in mice, the mice were irradiated with an 808 nm laser and the temperature of the mice before and after irradiation was monitored with an infrared camera. The results showed that the tumor temperature of mice in the BBPQDs group was up to 58.1 °C, which is significantly higher than that in the BPQDs group (~ 40.0 °C) and the PBS group (~ 33.6 °C) (Fig. 4a, b). The elevated temperature in the tumor region of the BBPQDs group may be due to the good tumor targeting, efficient accumulation and penetration of BBPQDs, which is consistent with the in vivo distribution results. The excellent photothermal properties of BBPQDs pave the way for subsequent in vivo antitumor applications.

### In vivo DCs activation

Next, we used flow cytometry to study the status of DCs in vivo. DCs are the most important antigen-presenting cells and play an important role in initiating, regulating and adapting to immunity. Once tumor antigens are exposed, immature DCs will capture and digest them, migrating them to the lymph nodes. Upon arrival at the lymph node, these immature DCs will mature and present major histocompatibility complex peptides to the T cell receptor. Therefore, the induction of more DCs maturation is important to enhance the efficacy of tumor immunotherapy [44, 45]. It has been reported that PTT is able to promote DCs maturation and induce tumor-specific immune responses by producing tumor-associated antigens in the ablated tumor site [46, 47]. To analyze the expression of DCs in tumors after different treatments, tumor-draining lymph nodes from each group of mice were taken 2 d after the end of treatment and subjected to corresponding fluorescent staining (CD11c, CD80 and CD86) and flow cytometric detection. The results showed that the BBPQDs + NIR group could effectively induce DCs maturation (32.80%), which was significantly higher than that of PBS alone (9.96%) or the BPQDs + NIR group (16.71%) (Fig. 4c, d). In summary, BBPQDs-mediated PTT could produce a stronger immunostimulatory effect, thus contributing to enhanced immunotherapy.

### Effects of combination therapy on primary tumors

**Fig. 5:**
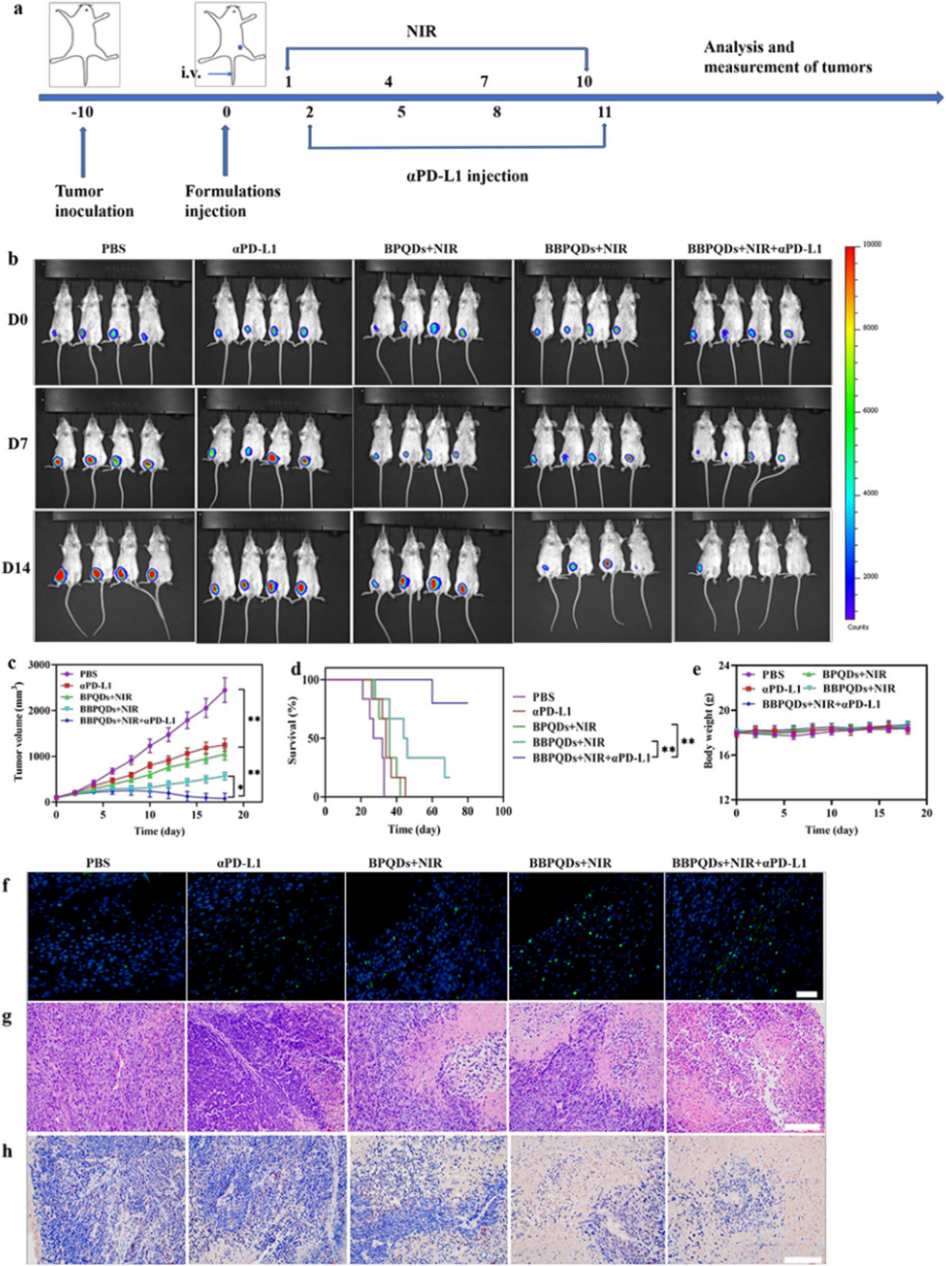
The therapeutic effect on primary tumors with photothermal immunotherapy strategy. **a** Schematic illustration of the animal experimental design for primary tumors. **b** Typical in vivo bioluminescence images of tumor burdens on day 0, day 7 and day 14. **c** The primary tumor growth curves under various treatments. **d** Survival curves of tumor-bearing mice after various treatments. **e** Body weight profiles of tumor-bearing mice during treatment. Representative fluorescent TUNEL (**f**) and H&E (**g**) stained images of tumor tissues in different treatment groups at the end of the experiments. Scale bar, 20 μm (TUNEL); scale bar, 100 μm (HE). **h** Immunohistochemical images showing the expression of Ki-67 in 4T1 tumor tissue. Scale bar, 100 μm

Encouraged by the excellent photothermal conversion efficiency and in vitro antitumor activity of BBPQDs + NIR, we further analyzed their tumor suppression efficiency in animal models. The flow chart of the in vivo experiments is shown in Fig. 5a. Briefly, a primary tumor model was established using BALB/c mice by subcutaneous injection of 4T1 cells to the left side of the mice. Ten days later, the primary tumor volume reached about 100 mm^3^ and the mice were randomly assigned into five different groups and treated with PBS; αPD-L1; BPQDs + NIR; BBPQDs + NIR; or BBPQDs + NIR + αPD-L1. Tumor growth was monitored by bioluminescence of 4T1-luc cells, and tumor size was also measured. Notably, BBPQDs + NIR-treated mice showed significant inhibition of tumor growth compared to the BPQDs + NIR group, suggesting that cancer cell membrane encapsulation-induced homologous targeting can be translated into antitumor response. Based on the tumor bioluminescence, tumor volume and harvested tumor weight, mice treated with BBPQDs + NIR + αPD-L1 showed the strongest antitumor effect (Fig. 5b, c, Additional file 1: Fig. S6–8S), which was due to the combined effect of BBPQDs-mediated PTT as well as αPD-L1 immunotherapy. In addition, limited antitumor effect was observed in those mice treated with αPD-L1. Treatment with BBPQDs + NIR + αPD-L1 resulted in a significant increase in survival time in mice (Fig. 5d). In addition, body weight growth was normal in all groups of mice, indicating that no toxicity occurred during treatment with the different agents (Fig. 5e).

**Fig. 6:**
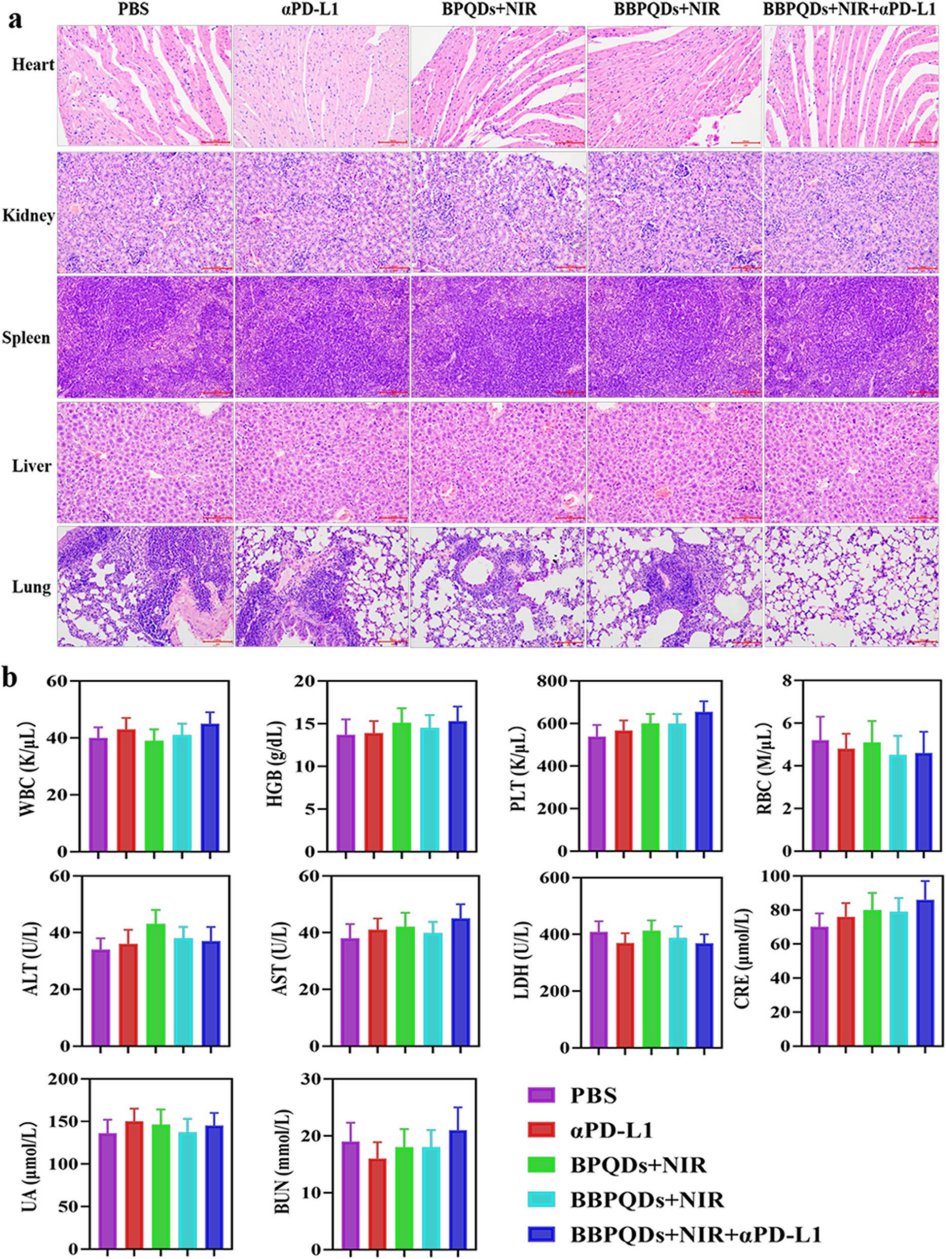
Biocompatibility evaluation of photothermal immunotherapy strategy.** a** H&E staining images of heart, kidney, spleen, liver and lung of mice under different treatments. Scale bars, 100 µm. **b** Blood count, liver function and renal function biomarkers

As a complement to the above efficacy evaluation, the tumor tissue samples were treated with TUNEL staining, which showed evidence that a large proportion of tumor cells in the BBPQDs + NIR + αPD-L1 group became apoptotic due to the combination treatment, while most of the tumor cells in the other groups survived (Fig. 5f). In addition, tumors were further used for histological examination by H&E and Ki-67 staining to investigate treatment-induced cell damage (Fig. 5g, h). In the BBPQDs + NIR + αPD-L1 group, a large number of fragments were detected all around the tumor cells, which is characteristic of PTT combined immunotherapy-induced tumor cell necrosis. In contrast, histological examination of the major organs (liver, spleen, lung, kidney, and heart) of the tumor-bearing mice revealed that the treatment groups did not cause significant toxic effects, clearly supporting their biocompatibility in vivo (Fig. 6a). It is also worth noting that metastases appeared in the lung tissues of all the groups except the BBPQDs + NIR + aPD-L1 group, which on the other hand confirms that the BBPQDs + NIR + aPD-L1 group has a better antitumor effect and can inhibit the metastasis of the tumors. To study the potential adverse effects of combined photothermal immunotherapy under clinically relevant conditions, we analyzed changes in blood cell counts, liver function, and kidney function (Fig. 6b). Compared with the control group, the BBPQDs + NIR + αPD-L1 group showed no significant abnormalities in the above blood indexes, thus indicating that the actual dosages provided good biocompatibility.

### Effects of combination therapy on distant tumors

**Fig. 7:**
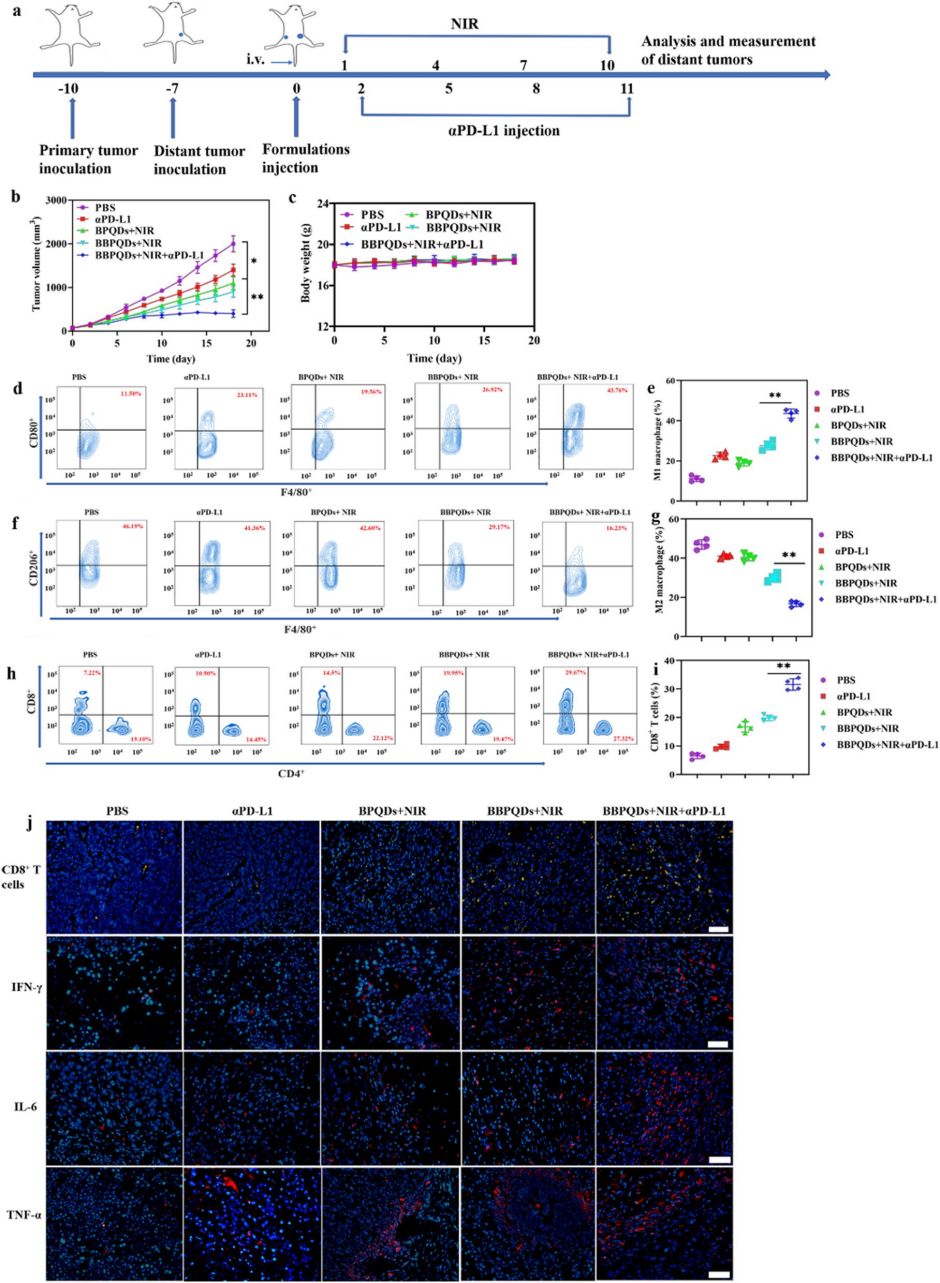
The therapeutic effect on abscopal tumors with the photothermal immunotherapy strategy. **a** Schematic illustration of the animal experimental design for abscopal tumors. **b** Abscopal tumor growth curves of the 4T1 tumor-bearing BALB/c mice model. **c** Body weight profiles of tumor-bearing mice during the treatment. Representative flow cytometry data (**d**) and statistical data (**e**) of CD80^+^ cells gated on CD11b^+^F4/80^+^ cells in the abscopal tumors. Representative flow cytometry data (**f**) and statistical data (**g**) of CD206^+^ cells gated on CD11b^+^F4/80^+^ cells in the abscopal tumors. Representative flow cytometry data (**h**) and statistical data (**i**) of CD8^+^ T cells in abscopal tumors (gated on CD3^+^ T cells).** j** Immunofluorescence staining of CD8^+^ T cells and cytokines (IFN-γ; IL-6; TNF-α) expressed in abscopal tumors. Scale bar, 20 μm

A bilateral subcutaneous 4T1 model was used to simulate primary and distant tumors. First, the primary tumor was inoculated to the left flank of the mice. Three days later, a second tumor was inoculated to the right flank to simulate the distant tumor (Fig. 7a). When the primary tumor volume was approximately 100 mm^3^, mice were randomly divided into five groups (n = 6 per group) and given PBS, αPD-L1, BPQDs + NIR, BBPQDs + NIR, and BBPQDs + NIR + αPD-L1. The primary tumors were irradiated with NIR; the distant tumors were not irradiated. Tumor volumes of distant tumors were measured. The results showed rapid distant tumor growth in the PBS group, while αPD-L1 alone, BPQDs + NIR, and BBPQDs + NIR exhibited only a moderate inhibitory effect on distant tumor growth (Fig. 7b, Additional file 1: Figs. S9, 10). Consistent with our above results, treatment with BBPQDs + NIR + αPD-L1 had a very significant inhibitory effect on the tumor growth, confirming that the combination therapy had a systemic antitumor effect, eliminating both the primary tumors and effectively inhibiting the growth of distant tumors. Furthermore, the body weights of the mice remained consistent across treatment groups, thereby indicating low systemic toxicity for this treatment modality (Fig. 7c).

We analyzed the TME and immune cells of abscopal tumors in mice to further investigate the mechanism of antitumor effects of the combination of BBPQDs-mediated PTT and αPD-L1 immunotherapy on distant tumors. Figure 7d, e show that the numbers of M1-like TAMs and the percentage of CD8^+^ T cells were significantly increased in the BBPQDs + NIR + αPD-L1 group, while M2-like TAMs (Fig. 7f, g) were significantly lower in distant tumors. The increased CD8^+^ T cells in the distant tumors can be attributed to local cross presentation of tumor antigens by macrophages and DCs that trigger systemic antitumor immunity (Fig. 7h, i). In addition, immunofluorescence assays further confirmed that the tumor-infiltrating CD8^+^ T cells were the most abundant in distant tumors in the BBPQDs + NIR + αPD-L1 group (Fig. 7j). In addition, the secretion of cytokines in distant tumors and in serum was also detected using CLSM and ELISA. As shown in Fig. 7j and Fig. S11, IFN-γ, IL-6 and TNF-α levels were the highest in distant tumor tissues and in serum of the BBPQDs + NIR + αPD-L1 group compared with other groups, thus demonstrating their enhanced antitumor effects. These results demonstrate that BBPQDs-based local PTT combined with αPD-L1 immunotherapy not only inhibits the growth of primary tumors at the irradiated site, but also suppresses the growth of unirradiated distant tumors due to the abscopal effect by regulating the immunosuppressive TME and promoting systemic antitumor immune response. These observations suggest that BBPQDs-based combination therapy has great potential as an effective strategy to treat cancer metastasis.

### Effects of combination therapy on rechallenged tumors

**Fig. 8:**
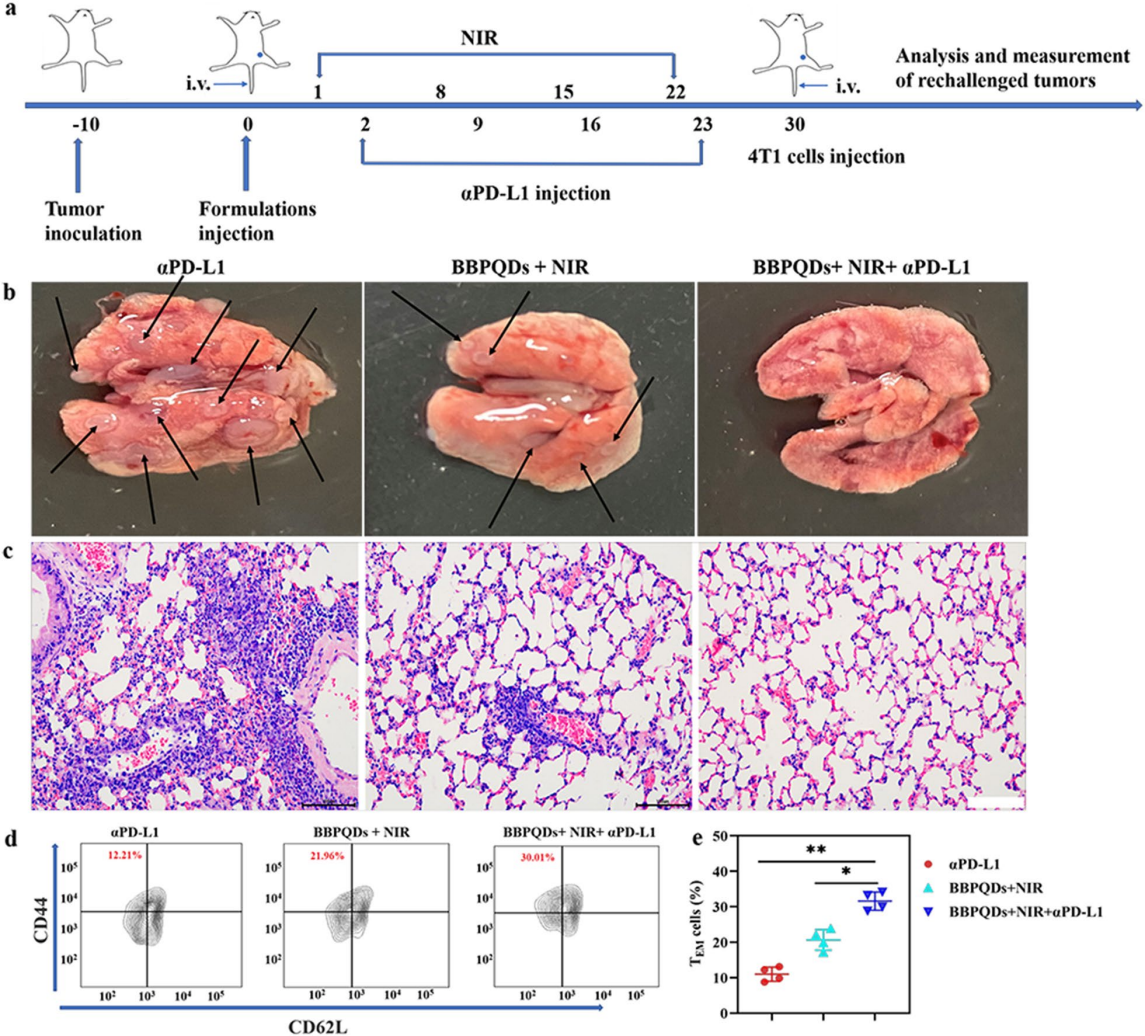
Metastasis prevention via photothermal immunotherapy strategy-induced long-term immune effects. **a** Schematic illustration for photothermal immunotherapy strategy-mediated inhibition of tumor metastasis. **b** Representative photographs of lung tissues with tumor metastasis. **c** H&E staining images of mice lungs under different treatments. Scale bars, 100 µm. Representative flow cytometry data (**d**) and statistical data (**e**) of T_EM_ in the spleen analyzed by flow cytometry (gated on CD3^+^CD8^+^ T cells) on day 33

Effector cells are rapidly produced and immune effects occur quickly when the body responds to the same antigen again due to the immune memory effect [48–50]. T_EM_ plays an important role in antitumor immune memory. We performed an immune memory experiment to confirm the immune memory effect of combination therapy of BBPQDs-mediated PTT and αPD-L1 treatment. The experimental design is shown in Fig. 8a. Mice were rechallenged with 4T1 cells in the tail vein 30 days after complete tumor removal by surgery or BBPQDs + NIR + αPD-L1. Mice were divided into three groups: (1) surgery + αPD-L1 group; (2) surgery + BBPQDs + NIR group; and (3) BBPQDs + NIR + αPD-L1 group. As shown in the Fig. 8b, c and Fig. S12, lung tumor metastases were obvious for primary tumors with surgery + αPD-L1 treatment, whereas BBPQDs mediated PTT had a significant inhibitory effect on the growth of rechallenged tumors, indicating that PTT can induce immune effects. The combined treatment strategy of BBPQDs-mediated PTT and αPD-L1 immunotherapy had the most significant tumor suppression ability, and almost no tumor metastases were observed in the lungs.

To investigate the mechanism of immune memory induction by the combination treatment, we examined the proportion of splenic T_EM_ (CD3^+^CD8^+^CD44^+^CD62L^−^) in mice using flow cytometry. T_EM_ was significantly higher in the BBPQDs + NIR + αPD-L1 group (31.59 ± 2.55%) compared to the αPD-L1 group (10.91 ± 1.99%) and BBPQDs + NIR group (20.66 ± 2.89%) (Fig. 8d, e). Results suggest that the combined treatment strategy of BBPQDs + NIR + αPD-L1 can not only eradicate the primary tumors and eliminate the distant tumors by triggering the systemic antitumor immune response through PTT, but also inhibit tumor recurrence and metastasis by generating an immune memory effect.

## Conclusions

TNBC has low effectiveness for ICB and is prone to recurrence and metastasis. Here, we constructed a combination therapy strategy based on BBPQDs-mediated PTT and αPD-L1 immunotherapy. BBPQDs can efficiently target localized tumors through both homologous targeting and tumor homing effects. Under NIR irradiation, the BBPQDs kill tumors directly through a photothermal effect, promote the maturation of DCs, and elicit activation of T cells to induce antitumor immune responses. The combination of PTT and αPD-L1 immunotherapy not only effectively treats distant tumors by reprograming the immunosuppressive TME, promoting local and systemic antitumor immune responses but also inhibits the tumor metastasis through the immune memory effect. This study provides a novel therapeutic concept for the treatment of TNBC with promising applications.

## Supplementary Information

**Additional file 1: Fig. S1.**Zeta potential of BPQDs, BBPQDs and cancer cell membrane. **Fig. S2**. The analysis of CD47, gp100 and Pan-Cadherin by Western blotting. A: cancer cell lysate; B: cancer cell membrane; and C: BBPQDs. **Fig. S3.** XRD spectra of the BPQDs and BBPQDs. **Fig. S4.** CLSM images of 4T1 cells after incubation with FITC labeled BPQDs and BBPQDs. Nuclei were stained with DAPI. **Fig. S5.** CLSM images of RAW 264.7 cells after incubation with FITC labeled BPQDs and BBPQDs. Nuclei were stained with DAPI. **Fig. S6.** Primary tumor growth curves of individual mice in different groups of the 4T1 tumor-bearing BALB/c mice model. A: PBS; B: αPD-L1; C: BPQDs+ NIR; D: BBPQDs+ NIR; and E: BBPQDs +NIR+ αPD-L1. **Fig. S7.** Tumor growth inhibition ratios of different groups on the primary tumors at the18th day of treatment. **Fig. S8.** Tumor weight of the sacrificed mice on the18th day of treatment. **Fig. S9.** Abscopal tumor growth curves of individual mice in different groups of the 4T1 tumor-bearing BALB/c mice model. A: PBS; B: αPD-L1; C: BPQDs+ NIR; D: BBPQDs+ NIR; and E: BBPQDs+ NIR+ αPD-L1. **Fig. S10.** Tumor growth inhibition ratios of different groups on the distant tumors on the 18th day of treatment. **Fig. S11.** Cytokine levels (IFN-γ, IL-6 and TNF-α) in serum from tumor-bearing mice isolated at 48 h after the last injection. **Fig. S12.** Quantification of pulmonary metastasis nodules in different groups of 4T1 tumor-bearing BALB/c mice.

## Data Availability

The data are available in the main manuscript, supplementary information files, and from the corresponding authors upon reasonable request.

## Abbreviations

TNBC: Triple-negative breast cancer
BBPQDs: Biomimetic black phosphorus quantum dots
ICB: Immune checkpoint blockade
TME: Tumor microenvironment
PTT: Photothermal therapy
NIR: Near-infrared
αPD-L1: Anti-PD-L1
PBS: Phosphate buffer solution
DAPI: 4′,6-Diamidino-2-phenylindole
DCs: Dendritic cells
H&E: Hematoxylin and eosin
IL-6: Interleukin-6
TNF-α: Tumor necrosis factor-α
IFN-γ: Interferon-γ
DLS: Dynamic light scattering
XRD: X-ray diffraction
CLSM: Confocal laser scanning microscopy
T_EM_: Effector memory T cells
RPMI: Roswell Park Memorial Institute
FBS: Fetal bovine serum
ELISA: Enzyme-linked immunosorbent assay
MTT: 3-(4,5-Dimethyl-2-thiazolyl)-2,5-diphenyl-2*H*-tetrazolium bromide
TEM: Transmission electron microscope
AFM: Atomic force microscope

## References

[1] Dawson SJ, Provenzano E, Caldas C (2009). Triple negative breast cancers: clinical and prognostic implications. Eur J Cancer.

[2] DeSantis CE, Fedewa SA, Goding Sauer A, Kramer JL, Smith RA, Jemal A (2016). Breast cancer statistics, 2015: convergence of incidence rates between black and white women. CA Cancer J Clin.

[3] Foulkes WD, Smith IE, Reis-Filho JS (2010). Triple-negative breast cancer. N Engl J Med.

[4] Dent R, Trudeau M, Pritchard KI, Hanna WM, Kahn HK, Sawka CA (2007). Triple-negative breast cancer: clinical features and patterns of recurrence. Clin Cancer Res.

[5] O'Reilly EA, Gubbins L, Sharma S, Tully R, Guang MH, Weiner-Gorzel K (2015). The fate of chemoresistance in triple negative breast cancer (TNBC). BBA Clin.

[6] Aysola K, Desai A, Welch C, Xu J, Qin Y, Reddy V (2013). Triple negative breast cancer—an overview. Hereditary Genet..

[7] Hudis CA, Gianni L (2011). Triple-negative breast cancer: an unmet medical need. Oncologist.

[8] Adams S, Schmid P, Rugo HS, Winer EP, Loirat D, Awada A (2019). Pembrolizumab monotherapy for previously treated metastatic triple-negative breast cancer: cohort A of the phase II KEYNOTE-086 study. Ann Oncol.

[9] Dirix LY, Takacs I, Jerusalem G, Nikolinakos P, Arkenau HT, Forero-Torres A (2018). Avelumab, an anti-PD-L1 antibody, in patients with locally advanced or metastatic breast cancer: a phase 1b JAVELIN Solid Tumor study. Breast Cancer Res Treat.

[10] Nanda R, Chow LQ, Dees EC, Berger R, Gupta S, Geva R (2016). Pembrolizumab in patients with advanced triple-negative breast cancer: phase Ib KEYNOTE-012 Study. J Clin Oncol.

[11] Bates JP, Derakhshandeh R, Jones L, Webb TJ (2018). Mechanisms of immune evasion in breast cancer. BMC Cancer.

[12] Liu Z, Li M, Jiang Z, Wang X (2018). A comprehensive immunologic portrait of triple-negative breast cancer. Transl Oncol.

[13] Chen Q, Xu L, Liang C, Wang C, Peng R, Liu Z (2016). Photothermal therapy with immune-adjuvant nanoparticles together with checkpoint blockade for effective cancer immunotherapy. Nat Commun.

[14] Li L, Yang S, Song L, Zeng Y, He T, Wang N (2018). An endogenous vaccine based on fluorophores and multivalent immunoadjuvants regulates tumor micro-environment for synergistic photothermal and immunotherapy. Theranostics.

[15] Ochoa de Olza M, Navarro Rodrigo B, Zimmermann S, Coukos G (2020). Turning up the heat on non-immunoreactive tumours: opportunities for clinical development. Lancet Oncol.

[16] Pan J, Wang Y, Zhang C, Wang X, Wang H, Wang J (2018). Antigen-directed fabrication of a multifunctional nanovaccine with ultrahigh antigen loading efficiency for tumor photothermal-immunotherapy. Adv Mater.

[17] Wang C, Xu L, Liang C, Xiang J, Peng R, Liu Z (2014). Immunological responses triggered by photothermal therapy with carbon nanotubes in combination with anti-CTLA-4 therapy to inhibit cancer metastasis. Adv Mater.

[18] Lal S, Clare SE, Halas NJ (2008). Nanoshell-enabled photothermal cancer therapy: impending clinical impact. Acc Chem Res.

[19] Melancon MP, Zhou M, Li C (2011). Cancer theranostics with near-infrared light-activatable multimodal nanoparticles. Acc Chem Res.

[20] von Maltzahn G, Park JH, Agrawal A, Bandaru NK, Das SK, Sailor MJ (2009). Computationally guided photothermal tumor therapy using long-circulating gold nanorod antennas. Cancer Res.

[21] Thakor AS, Gambhir SS (2013). Nanooncology: the future of cancer diagnosis and therapy. CA Cancer J Clin.

[22] Kong L, Yuan F, Huang P, Yan L, Cai Z, Lawson T (2020). A metal-polymer hybrid biomimetic system for use in the chemodynamic-enhanced photothermal therapy of cancers. Small.

[23] Sun H, Zhang Y, Chen S, Wang R, Chen Q, Li J (2020). Photothermal fenton nanocatalysts for synergetic cancer therapy in the second near-infrared window. ACS Appl Mater Interfaces.

[24] Gong B, Shen Y, Li H, Li X, Huan X, Zhou J (2021). Thermo-responsive polymer encapsulated gold nanorods for single continuous wave laser-induced photodynamic/photothermal tumor therapy. J Nanobiotechnology.

[25] Han R, Xiao Y, Yang Q, Pan M, Hao Y, He X (2021). Ag_2_S nanoparticle-mediated multiple ablations reinvigorates the immune response for enhanced cancer photo-immunotherapy. Biomaterials.

[26] Song J, Yang X, Jacobson O, Lin L, Huang P, Niu G (2015). Sequential drug release and enhanced photothermal and photoacoustic effect of hybrid reduced graphene oxide-loaded ultrasmall gold nanorod vesicles for cancer therapy. ACS Nano.

[27] Song J, Yang X, Yang Z, Lin L, Liu Y, Zhou Z (2017). Rational design of branched nanoporous gold nanoshells with enhanced physico-optical properties for optical imaging and cancer therapy. ACS Nano.

[28] Xie X, Gao W, Hao J, Wu J, Cai X, Zheng Y (2021). Self-synergistic effect of Prussian blue nanoparticles for cancer therapy: driving photothermal therapy and reducing hyperthermia-induced side effects. J Nanobiotechnology.

[29] Li B, Lai C, Zeng G, Huang D, Qin L, Zhang M (2019). Black phosphorus, a rising star 2D nanomaterial in the post-graphene era: synthesis, properties, modifications, and photocatalysis applications. Small.

[30] Xing B, Guan L, Yu Y, Niu X, Yan X, Zhang S (2019). HfO_2_-passivated black phosphorus field effect transistor with long-termed stability and enhanced current on/off ratio. Nanotechnology.

[31] Sun Z, Xie H, Tang S, Yu XF, Guo Z, Shao J (2015). Ultrasmall black phosphorus quantum dots: synthesis and use as photothermal agents. Angew Chem Int Ed Engl.

[32] Sun C, Wen L, Zeng J, Wang Y, Sun Q, Deng L (2016). One-pot solventless preparation of PEGylated black phosphorus nanoparticles for photoacoustic imaging and photothermal therapy of cancer. Biomaterials.

[33] Jiang Y, Krishnan N, Zhou J, Chekuri S, Wei X, Kroll AV (2020). Engineered cell-membrane-coated nanoparticles directly present tumor antigens to promote anticancer immunity. Adv Mater.

[34] Chen Z, Zhao P, Luo Z, Zheng M, Tian H, Gong P (2016). Cancer cell membrane-biomimetic nanoparticles for homologous-targeting dual-modal imaging and photothermal therapy. ACS Nano.

[35] Rao L, Bu LL, Cai B, Xu JH, Li A, Zhang WF (2016). Cancer cell membrane-coated upconversion nanoprobes for highly specific tumor imaging. Adv Mater.

[36] Shao D, Zhang F, Chen F, Zheng X, Hu H, Yang C (2020). Biomimetic diselenide-bridged mesoporous organosilica nanoparticles as an X-ray-responsive biodegradable carrier for chemo-immunotherapy. Adv Mater.

[37] Li S, Jiang W, Yuan Y, Sui M, Yang Y, Huang L (2020). Delicately designed cancer cell membrane-camouflaged nanoparticles for targeted (19)F MR/PA/FL imaging-guided photothermal therapy. ACS Appl Mater Interfaces.

[38] Sun Z, Zhao Y, Li Z, Cui H, Zhou Y, Li W (2017). TiL4-coordinated black phosphorus quantum dots as an efficient contrast agent for in vivo photoacoustic imaging of cancer. Small.

[39] Zhao P, Qiu L, Zhou S, Li L, Qian Z, Zhang H (2021). Cancer cell membrane camouflaged mesoporous silica nanoparticles combined with immune checkpoint blockade for regulating tumor microenvironment and enhancing antitumor therapy. Int J Nanomedicine.

[40] Sun P, Zhou D, Gan Z (2011). Novel reduction-sensitive micelles for triggered intracellular drug release. J Control Release.

[41] Zhao P, Li L, Zhou S, Qiu L, Qian Z, Liu X (2018). TPGS functionalized mesoporous silica nanoparticles for anticancer drug delivery to overcome multidrug resistance. Mater Sci Eng C Mater Biol Appl.

[42] Huang S, Ling X (2017). Black phosphorus: optical characterization, properties and applications. Small.

[43] Huang LL, Nie W, Zhang J, Xie HY (2020). Cell-membrane-based biomimetic systems with bioorthogonal functionalities. Acc Chem Res.

[44] Huh JC, Strickland DH, Jahnsen FL, Turner DJ, Thomas JA, Napoli S (2003). Bidirectional interactions between antigen-bearing respiratory tract dendritic cells (DCs) and T cells precede the late phase reaction in experimental asthma: DC activation occurs in the airway mucosa but not in the lung parenchyma. J Exp Med.

[45] Janeway CA, Bottomly K (1994). Signals and signs for lymphocyte responses. Cell.

[46] Chen WR, Liu H, Ritchey JW, Bartels KE, Lucroy MD, Nordquist RE (2002). Effect of different components of laser immunotherapy in treatment of metastatic tumors in rats. Cancer Res.

[47] Wang T, Wang D, Yu H, Feng B, Zhou F, Zhang H (2018). A cancer vaccine-mediated postoperative immunotherapy for recurrent and metastatic tumors. Nat Commun.

[48] D'Souza WN, Hedrick SM (2006). Cutting edge: latecomer CD8 T cells are imprinted with a unique differentiation program. J Immunol.

[49] Kinjyo I, Qin J, Tan SY, Wellard CJ, Mrass P, Ritchie W (2015). Real-time tracking of cell cycle progression during CD8+ effector and memory T-cell differentiation. Nat Commun.

[50] Teixeiro E, Daniels MA, Hamilton SE, Schrum AG, Bragado R, Jameson SC (2009). Different T cell receptor signals determine CD8+ memory versus effector development. Science.

