# Supplementary material for: Biomimetic black phosphorus quantum dots-based photothermal therapy combined with anti-PD-L1 treatment inhibits recurrence and metastasis in triple-negative breast cancer
Source: J Nanobiotechnology. 2021 Jun 13;19:181. doi: 10.1186/s12951-021-00932-2 (PMC8201856; doi:10.1186/s12951-021-00932-2)
Supplement: Supplementary file 1 — Additional file 1: Fig. S1.Zeta potential of BPQDs, BBPQDs and cancer cell membrane. Fig. S2. The analysis of CD47, gp100 and Pan-Cadherin by Western blotting. A: cancer cell lysate; B: cancer cell membrane; and C: BBPQDs. Fig. S3. XRD spectra of the BPQDs and BBPQDs. Fig. S4. CLSM images of 4T1 cells after incubation with FITC labeled BPQDs and BBPQDs. Nuclei were stained with DAPI. Fig. S5. CLSM images of RAW 264.7 cells after incubation with FITC labeled BPQDs and BBPQDs. Nuclei were stained with DAPI. Fig. S6. Primary tumor growth curves of individual mice in different groups of the 4T1 tumor-bearing BALB/c mice model. A: PBS; B: αPD-L1; C: BPQDs+ NIR; D: BBPQDs+ NIR; and E: BBPQDs +NIR+ αPD-L1. Fig. S7. Tumor growth inhibition ratios of different groups on the primary tumors at the18th day of treatment. Fig. S8. Tumor weight of the sacrificed mice on the18th day of treatment. Fig. S9. Abscopal tumor growth curves of individual mice in different groups of the 4T1 tumor-bearing BALB/c mice model. A: PBS; B: αPD-L1; C: BPQDs+ NIR; D: BBPQDs+ NIR; and E: BBPQDs+ NIR+ αPD-L1. Fig. S10. Tumor growth inhibition ratios of different groups on the distant tumors on the 18th day of treatment. Fig. S11. Cytokine levels (IFN-γ, IL-6 and TNF-α) in serum from tumor-bearing mice isolated at 48 h after the last injection. Fig. S12. Quantification of pulmonary metastasis nodules in different groups of 4T1 tumor-bearing BALB/c mice. [file 12951_2021_932_MOESM1_ESM.docx]

**Additional file 1**

Biomimetic black phosphorus quantum dots-based photothermal therapy combined with anti-PD-L1 treatment inhibits recurrence and metastasis in triple-negative breast cancer

Peiqi Zhao^1#*^, Yuanlin Xu^2#^, Wei Ji^3^, Shiyong Zhou^1^, Lanfang Li^1^, Lihua Qiu^1^, Zhengzi Qian^1^, Xianhuo Wang^1^, Huilai Zhang^1*^

1 Department of Lymphoma, Tianjin’s Clinical Research Center for Cancer, Key Laboratory of Cancer Prevention and Therapy, National Clinical Research Center for Cancer, Tianjin Medical University Cancer Institute and Hospital, Tianjin Medical University, Tianjin, 300060, China.

2 Department of Lymphatic Comprehensive Internal Medicine, Affiliated Cancer Hospital of Zhengzhou University, Zhengzhou, 450001, Henan, China.

3 Public Laboratory, Tianjin’s Clinical Research Center for Cancer, Key Laboratory of Cancer Prevention and Therapy, National Clinical Research Center for Cancer, Tianjin Medical University Cancer Institute and Hospital, Tianjin Medical University, Tianjin, 300060, China.

^#^These authors contributed equally to this work.

^*^Corresponding author: [peiqizhao@126.com](mailto:peiqizhao@126.com); [zhanghltch@163.com](mailto:zhanghltch@163.com)

**Materials and Methods**

**Materials**

The bulk BP crystals were purchased from MOPHOS (China) and stored in a dark vacuum glovebox. Roswell Park Memorial Institute (RPMI) 1640 medium and fetal bovine serum (FBS) were purchased from Gibco Life Technologies (USA). The antibodies of CD3, CD4, CD8, CD11b, CD11c, CD44, CD45, CD62L, CD80, CD86, CD206 and F4/80 and the enzyme-linked immunosorbent assay (ELISA) test kits were acquired from Sigma-Aldrich (USA). 3-(4,5-dimethyl-2-thiazolyl)-2,5-diphenyl-2H-tetrazolium bromide (MTT) assay and fluorescein isothiocyanate (FITC) were purchased from Thermo Fisher Scientific (USA). The Annexin V-FITC/PI Apoptosis Detection Kit and TUNEL Apoptosis Detection Kit were obtained from Vazyme (China). The Cy5.5-NHS was purchased from Lumiprobe (USA). αPD-L1 was obtained from Bio X Cell (USA). All other reagents were analytically pure and used directly without further purification.

4T1 (triple negative breast cancer cell line), 4T1-luc, HBL-100 (human mammary epithelial cell line) and RAW 264.7 (mouse macrophage cell line) cells were bought from American Type Culture Collection (Manassas, VA, USA). The cells were cultured in a RPMI 1640 medium containing 10% FBS, penicillin (100 UI/mL), and streptomycin (100 UI/mL) with 5% CO_2_ at 37 °C.

BALB/c mice (5-6 weeks old) were obtained by SiPeiFu Biotechnology Co., Ltd (Beijing, China). All animal experiments were approved by the Animal Ethical and Welfare Committee of Tianjin Medical University Cancer Institute and Hospital, and all animal studies were conducted in accordance with the guidelines of this committee.

**Synthesis of BPQDs**

The BPQDs were prepared from bulk BP by sonication assisted liquid-phase exfoliation. Briefly, 25 mg BP was dispersed in 50 mL N-methyl-2-pyrrolidinone (1 mg/mL). The mixture was sonicated in an ice bath with an ultrasonic cell disruption system (1200 W, with probe) for 3 h, followed by ultrasonic cleaning for 10 h (300 W). Oversized bulk BP was then removed by centrifugation (7000 rpm, 20 min). The suspension was further centrifuged (13,000 rpm, 20 min) and the precipitate was rinsed with deionized water and freeze-dried under vacuum.

**Preparation of cancer cell membrane**

Cancer cell membranes were firstly prepared using a previous method [1]. Briefly, the collected 4T1 cells were resuspended in hypotonic lysis buffer containing membrane protein extraction reagent and incubated in an ice bath for 10 min, allowing freeze-thaw lysis of the cells in solution, followed by centrifugation (1000 g, 7 min) at 4 °C. To remove excess cancer cell vesicles and collect cell membrane debris, the supernatant was further centrifuged (14,000 g, 30 min). We then extruded the samples11 times using an Avanti mini-extruder. Finally, we centrifuged to remove the excess cancer cell vesicles.

**Physicochemical characterization**

The morphologies of BPQDs and BBPQDs were observed by a transmission electron microscope (TEM) (Carl Zeiss, Germany) and atomic force microscope (AFM) (FSM-Precision, China). The diameter and zeta potential of the BPQDs and BBPQDs were detected using a Zetasizer Nano (Malven, UK). Raman spectra were obtained by Raman spectrometer (Derbyshire, UK). The crystalline structures of the BPQDs and BBPQDs were investigated by the X-ray diffraction (XRD, Bruker, Japan). The in vivo biodistribution of BPQDs and BBPQDs in mice was investigated using the IVIS in vivo imaging system (PerkinElmer, USA). Flow cytometric analysis was performed with a BD LSRFortessa (BD Bioscience, USA).

**Western blotting**

Membrane proteins of cell lysates, cell membrane fragments, and BBPQDs were first extracted, and the total proteins were measured using the BCA assay kit (Beyotime, China). The proteins were separated by sodium dodecyl sulfate polyacrylamide gel electrophoresis and then transferred to polyvinylidene fluoride membranes. The membranes were blocked with 5% fat-free milk and will be incubated with primary antibody overnight at 4°C. The membranes were then incubated with horseradish peroxidase-conjugated secondary antibodies. Specific proteins were detected using an enhanced chemiluminescence detection assay (Santa Cruz Biotechnology, Inc.).

**Biocompatibility experiment**

To determine the in vitro cytotoxicity of BPQDs and BBPQDs, HBL-100 and 4T1 cells (1×10^4^ cells per well) were co-cultured with different concentrations of BPQDs and BBPQDs for 24 h, and then the cell viability was measured with a MTT assay according to the manufacturer’s protocol.

**Cellular uptake experiment**

4T1 and RAW 264.7 cells were inoculated in a 12-well plate at a density of 1 × 10^5^ cells and incubated overnight. The FITC labeled BPQDs and BBPQDs dispersed in RPMI-1640 medium were then added, allowing the cells to incubate for another 4 h. Subsequently, the medium was removed and washed three times with ice-cold PBS solution. Next, the nuclei were labeled with DAPI. Finally, the cells were observed using confocal laser scanning microscopy (CLSM, Leica, GER).


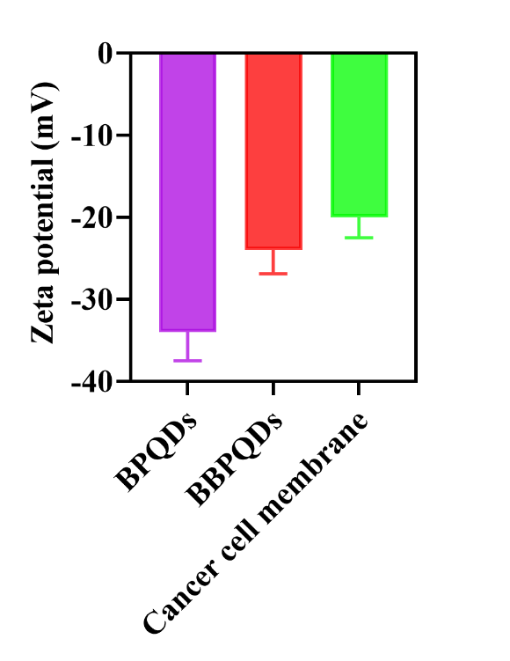


Fig. S1. Zeta potential of BPQDs, BBPQDs and cancer cell membrane.


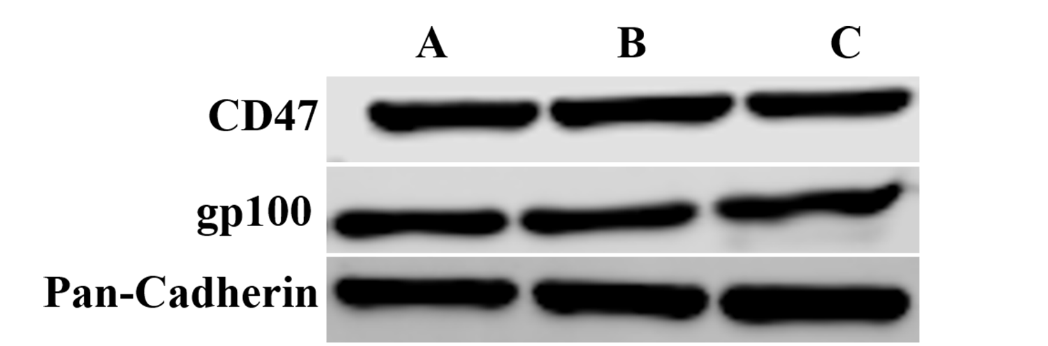


Fig. S2. The analysis of CD47, gp100 and Pan-Cadherin by Western blotting. A: cancer cell lysate; B: cancer cell membrane; and C: BBPQDs.


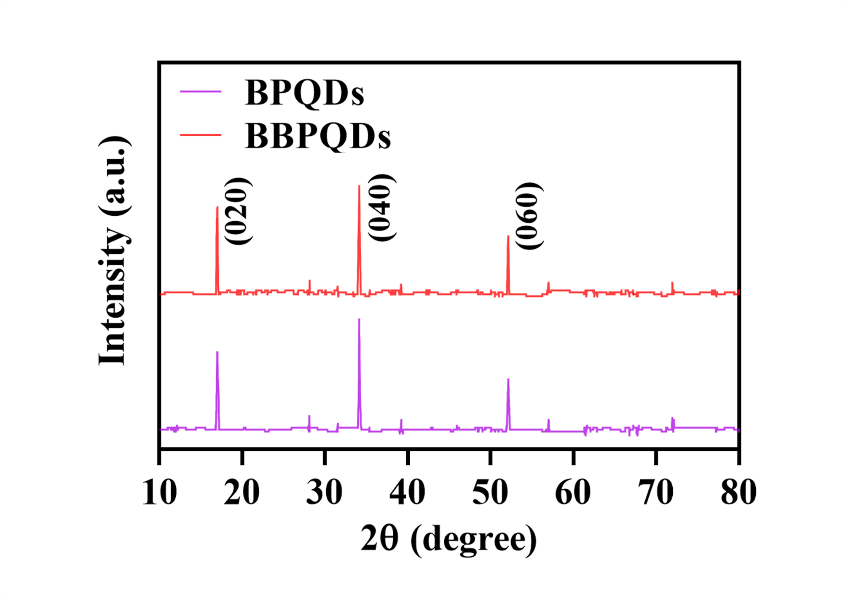


Fig. S3. XRD spectra of the BPQDs and BBPQDs.


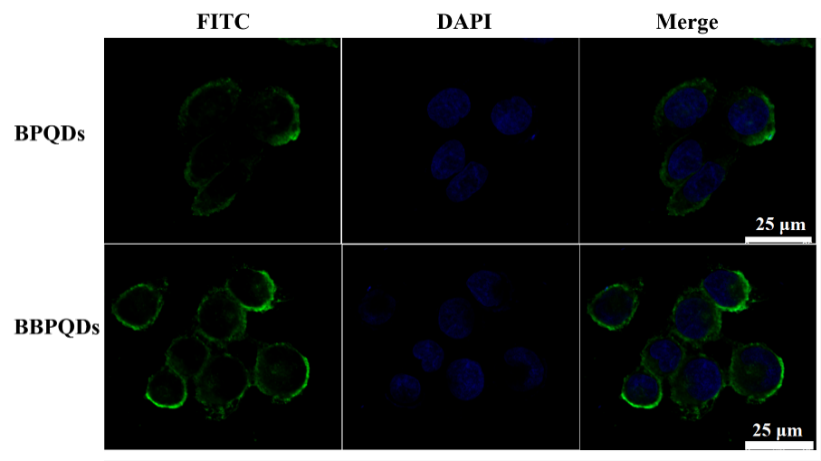


Fig. S4. CLSM images of 4T1 cells after incubation with FITC labeled BPQDs and BBPQDs. Nuclei were stained with DAPI.


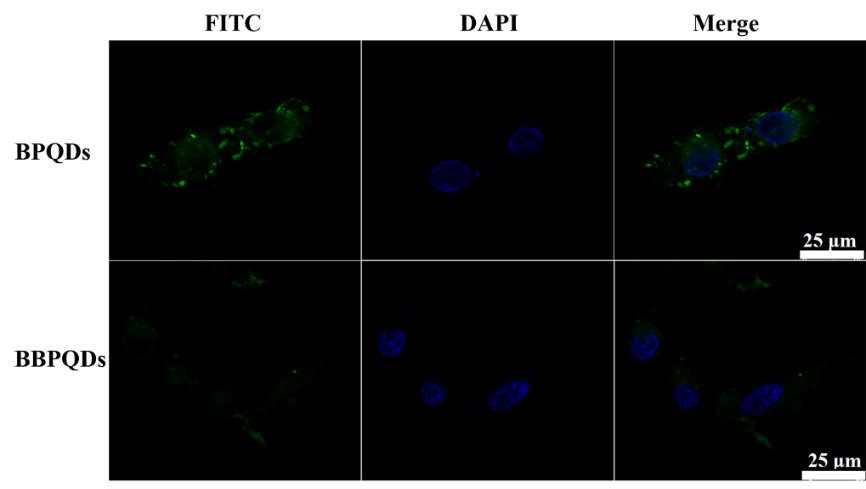


Fig. S5. CLSM images of RAW 264.7 cells after incubation with FITC labeled BPQDs and BBPQDs. Nuclei were stained with DAPI.


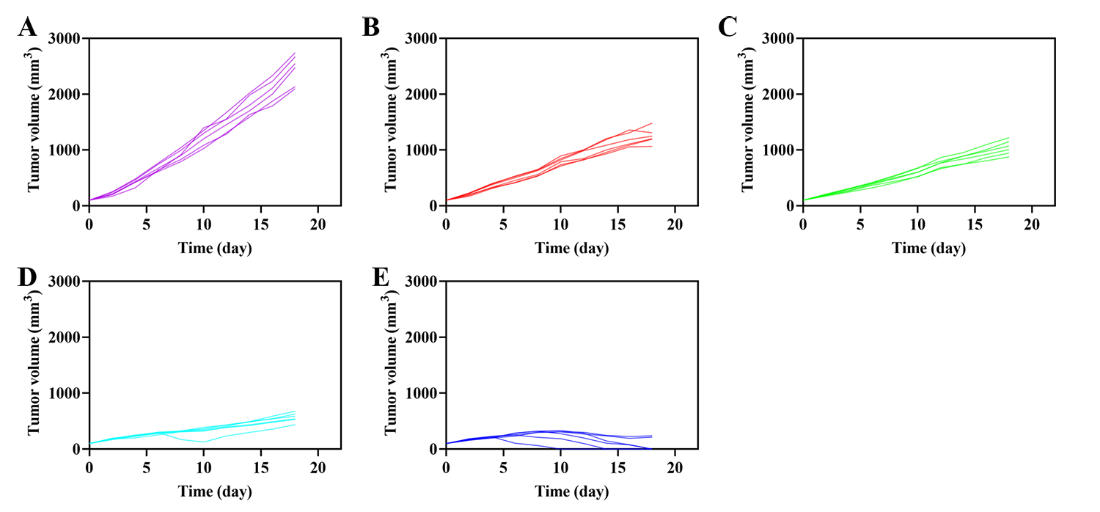


Fig. S6. Primary tumor growth curves of individual mice in different groups of the 4T1 tumor-bearing BALB/c mice model. A: PBS; B: αPD-L1; C: BPQDs+ NIR; D: BBPQDs+ NIR; and E: BBPQDs +NIR+ αPD-L1.


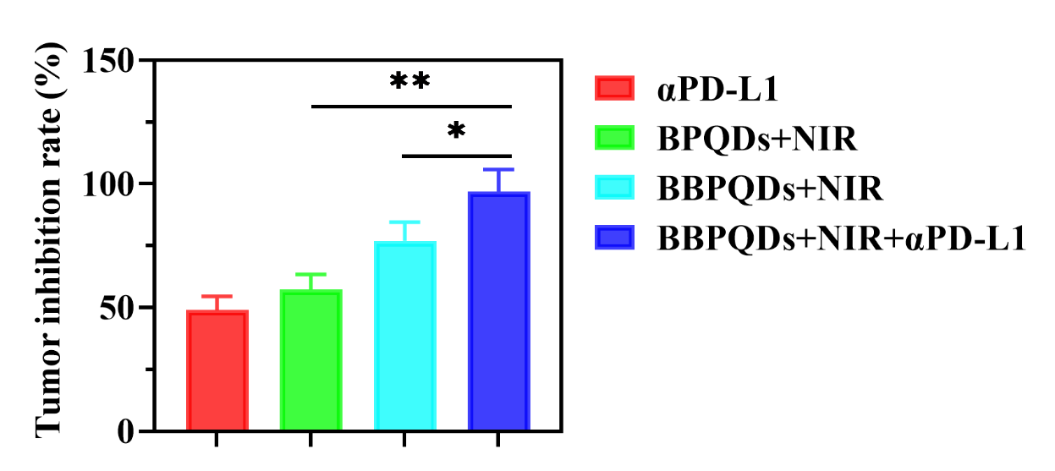


Fig. S7. Tumor growth inhibition ratios of different groups on the primary tumors at the18th day of treatment.


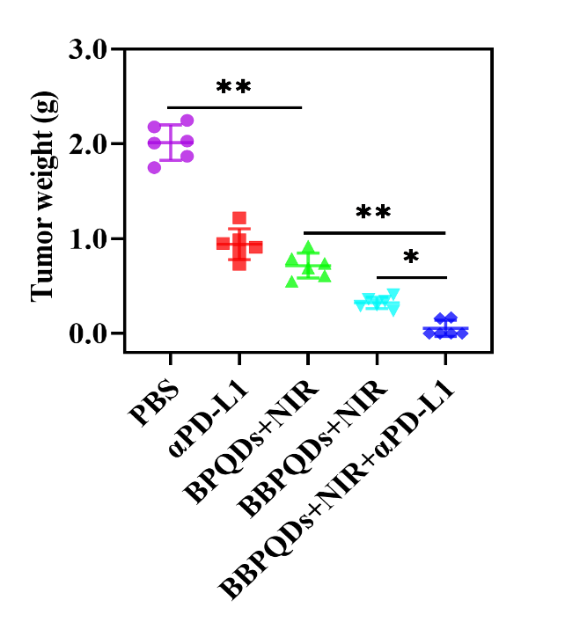


Fig. S8. Tumor weight of the sacrificed mice on the18th day of treatment.


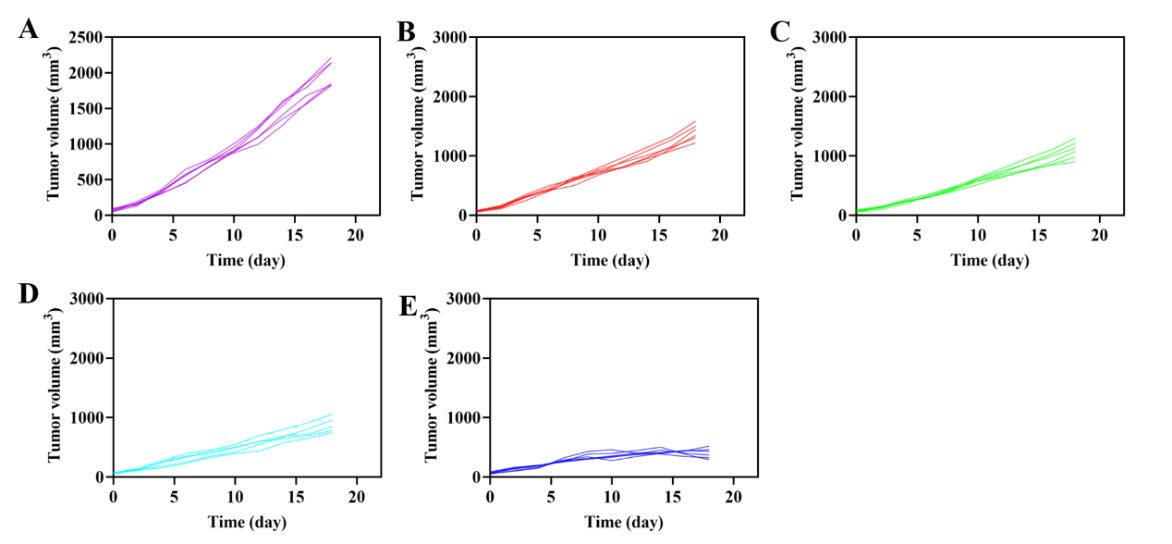


Fig. S9. Abscopal tumor growth curves of individual mice in different groups of the 4T1 tumor-bearing BALB/c mice model. A: PBS; B: αPD-L1; C: BPQDs+ NIR; D: BBPQDs+ NIR; and E: BBPQDs+ NIR+ αPD-L1.


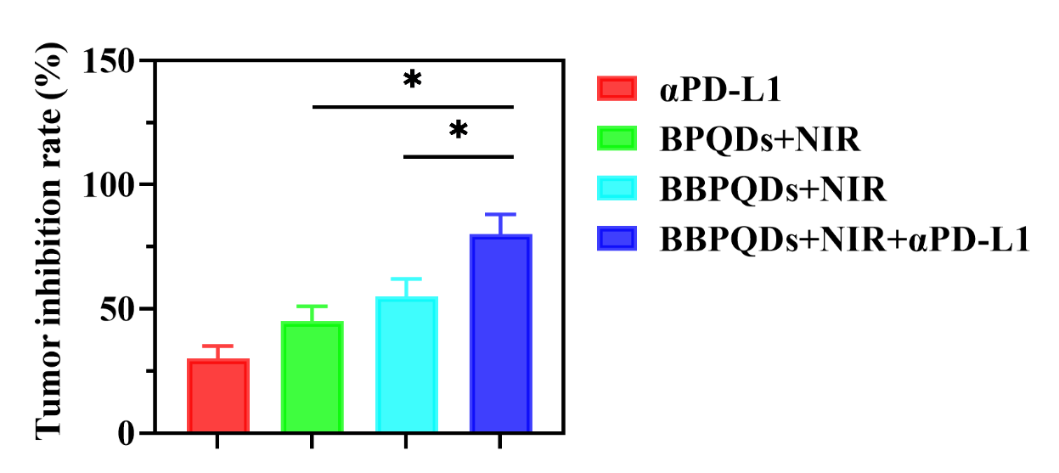


Fig. S10. Tumor growth inhibition ratios of different groups on the distant tumors on the 18th day of treatment.


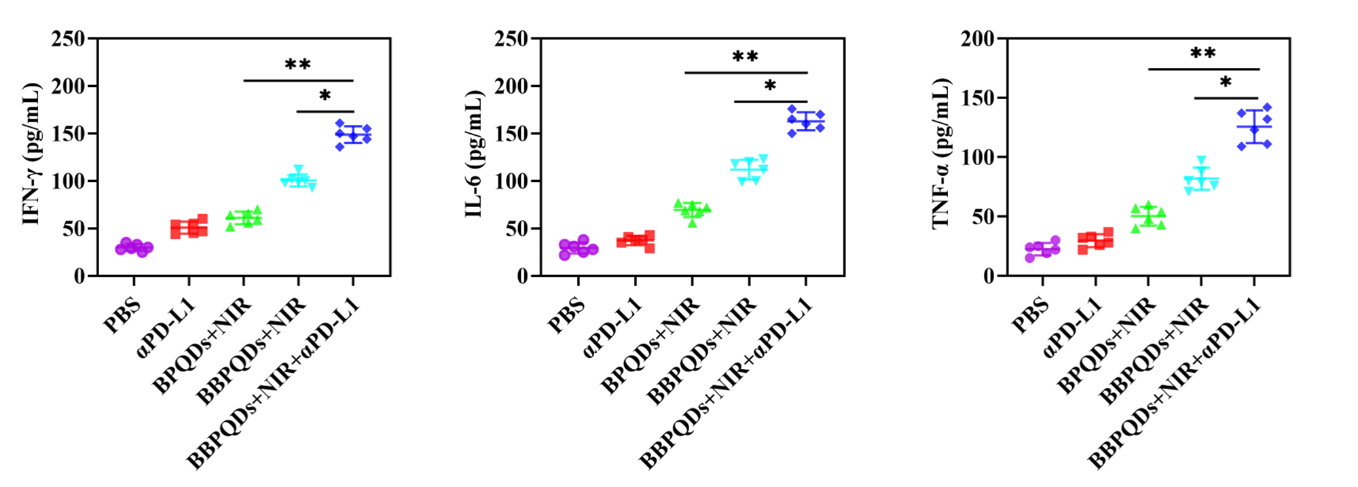


Fig. S11. Cytokine levels (IFN-γ, IL-6 and TNF-α) in serum from tumor-bearing mice isolated at 48 h after the last injection.


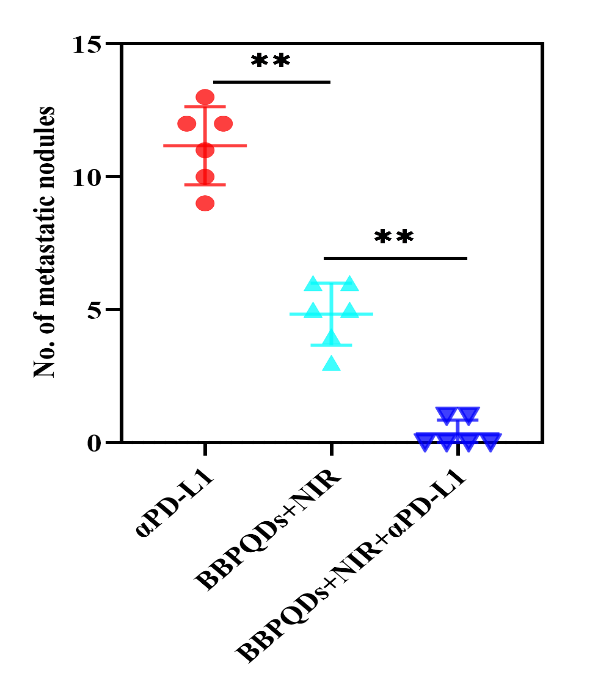


Fig. S12. Quantification of pulmonary metastasis nodules in different groups of 4T1 tumor-bearing BALB/c mice.

**Reference**

1. Zhao P, Qiu L, Zhou S, Li L, Qian Z, Zhang H. Cancer cell membrane camouflaged mesoporous silica nanoparticles combined with immune checkpoint blockade for regulating tumor microenvironment and enhancing antitumor therapy. Int J Nanomedicine. 2021;16:2107-2121.
